# Construction of the influenza A virus infection-induced cell-specific inflammatory regulatory network based on mutual information and optimization

**DOI:** 10.1186/1752-0509-7-105

**Published:** 2013-10-20

**Authors:** Suoqin Jin, Xiufen Zou

**Affiliations:** 1 School of Mathematics and Statistics, Wuhan University, Wuhan 430072, China

## Abstract

**Background:**

Influenza A virus (IAV) infection-induced inflammatory regulatory networks (IRNs) are extremely complex and dynamic. Specific biological experiments for investigating the interactions between individual inflammatory factors cannot provide a detailed and insightful multidimensional view of IRNs. Recently, data from high-throughput technologies have permitted system-level analyses. The construction of large and cell-specific IRNs from high-throughput data is essential to understanding the pathogenesis of IAV infection.

**Results:**

In this study, we proposed a computational method, which combines nonlinear ordinary differential equation (ODE)-based optimization with mutual information, to construct a cell-specific optimized IRN during IAV infection by integrating gene expression data with a prior knowledge of network topology. Moreover, we used the average relative error and sensitivity analysis to evaluate the effectiveness of our proposed approach. Furthermore, from the optimized IRN, we confirmed 45 interactions between proteins in biological experiments and identified 37 new regulatory interactions and 8 false positive interactions, including the following interactions: IL1β regulates TLR3, TLR3 regulates IFN-β and TNF regulates IL6. Most of these regulatory interactions are statistically significant by Z-statistic. The functional annotations of the optimized IRN demonstrated clearly that the defense response, immune response, response to wounding and regulation of cytokine production are the pivotal processes of IAV-induced inflammatory response. The pathway analysis results from the Kyoto Encyclopaedia of Genes and Genomes (KEGG) showed that 8 pathways are enriched significantly. The 5 pathways were validated by experiments, and 3 other pathways, including the intestinal immune network for IgA production, the cytosolic DNA-sensing pathway and the allograft rejection pathway, are the predicted novel pathways involved in the inflammatory response.

**Conclusions:**

Integration of knowledge-driven and data-driven methods allows us to construct an effective IRN during IAV infection. Based on the constructed network, we have identified new interactions among inflammatory factors and biological pathways. These findings provide new insight into our understanding of the molecular mechanisms in the inflammatory network in response to IAV infection. Further characterization and experimental validation of the interaction mechanisms identified from this study may lead to a novel therapeutic strategy for the control of infections and inflammatory responses.

## Background

Influenza A virus (IAV) infection is a worldwide public health threat [1,2]. IAV causes respiratory tract infections and leads to inflammatory responses. Controlling the inflammatory response resulting from an IAV infection is of great significance in reducing associated tissue damage. However, many biological experiments have demonstrated that IAV infection-induced inflammatory responses are extremely complicated and regulated by dynamic networks [3-5]. Specific biological experiments investigating the mechanisms of interactions among individual inflammatory factors have not provided a sufficiently detailed and insightful multidimensional view of inflammatory regulatory networks (IRNs). We need to investigate the mechanisms at a system-level and from the network dynamics. Therefore, the construction of large and cell-specific inflammatory regulatory networks (IRNs) based on high-throughput data is essential for investigating the molecular mechanisms of inflammatory responses during IAV infection.

Biological experiments have found that IAVs induce the expression of a number of inflammatory molecules and inflammatory cytokines and chemokines, such as IL27, IL32, IL6, TNF, IFNG, CXCL10, CCL3, NOS2 and IL8 [6-9]. Furthermore, a number of studies have shown that the H5N1 viruses can induce increased gene transcription of pro-inflammatory cytokines, including CXCL10, IFN-β, IL6, COX-2 (Cyclooxygenase-2) and CCL5 [9-12]. In particular, COX-2 is the primary mediator in protection against IAV infection [4] and has been shown to play a regulatory role in the induction of the H5N1-mediated pro-inflammatory cascade [10,11]. It is important to further investigate the mechanisms of the inflammatory cascade downstream of COX-2 regulation that may be involved in H5N1 infection [13]. To our best knowledge, the studies on constructing a cell-specific IRN after IAV infection are limited, and an integrated and systematic analysis of the inflammatory cascade mediated by COX-2 that incorporates microarray data has not yet been reported.

A number of different methods for inferring gene regulatory networks (GRNs) from high-throughput data have been proposed [14-20]. However, there are a few studies on the construction of dynamic signaling networks based on stoichiometric approaches, discrete Boolean models, the fuzzy logic models, the integer programming method and the ordinary differential equation (ODE)-based method [15,21-27]. No study has reported combining a prior knowledge of network topology with nonlinear optimization algorithms to identify the dynamic regulatory network. In the process of reconstructing networks from expression data based on a priori knowledge of network topology, the most important steps are converting familiar network maps into mathematical models and fitting the available data into the network’s structural parameters. Recently, the rough topological structure of inflammatory networks with 2361 nodes and 63276 edges in humans have been obtained, which provides a prelude to more detailed network analysis and mathematical modeling for an inflammatory network [28]. By combining information theory-based MI and nonlinear ODE-based optimization, in this study, we proposed a computational method to construct a cell-specific IRN mediated by COX-2 during IAV infection. A differential evolution (DE) algorithm was used to optimize the network so that it best fits the experimental data. Furthermore, we performed a Kyoto Encyclopaedia of Genes and Genomes (KEGG) pathway and gene ontology (GO) terms enrichment analysis on the optimized IRN to identify the underlying mechanisms during IAV infection.

## Methods

**Figure 1:**
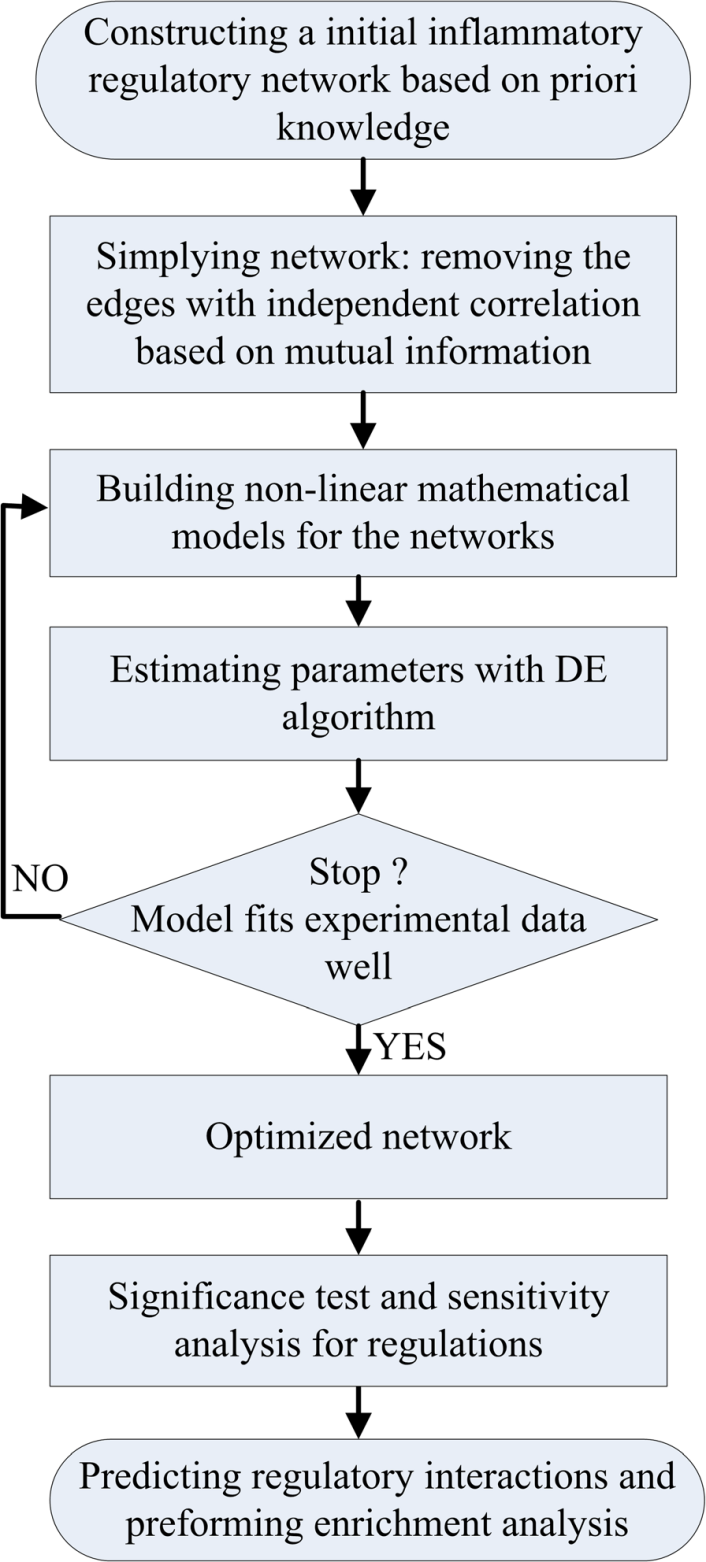
The flowchart of our work.

The flowchart of our work is presented in Figure 1 and mainly consists of six steps: constructing an initial IRN, simplifying the initial network, building a mathematical model, estimating parameters in the model with the DE algorithm, significance test and sensitivity analysis for the regulations, and performing an enrichment analysis.

### Data collection and construction of the initial inflammatory regulatory network

To construct a cell-specific IRN and investigate the mechanisms of the inflammatory cascade mediated by COX-2 in IAV infection, we selected 59 proteins, which are listed in Table 1, that are associated with the inflammatory responses regulated by COX-2 based on the published literature [3,9,13,29,30]. The microarray data were retrieved from the Gene Expression Omnibus (GEO) database under the GEO accession number GSE28166 [31,32]. This dataset contains 36 samples in total, with 3 mock and infected replicates for each time point. In this study, the expression levels of complexes were the average of the gene expression levels of the members of the complex from the dataset. The expression level of IAV was obtained from the literature [32].

**Table 1:** Total 59 proteins selected from the published literatures

**Gene symbol**	**Description**	**Type**
BPIFA1	BPI fold containing family A, member 1	Other
CCL16	Chemokine ligand 16	Cytokine
CCL2	Chemokine (C-C motif) ligand 2	Cytokine
CCL22	Chemokine (C-C motif) ligand 22	Cytokine
CCL5	Chemokine (C-C motif) ligand 5	Cytokine
CCR9	Chemokine (C-C motif) receptor 9	G-protein coupled receptor
CD14	CD14 molecule	Transmembrane receptor
CD27	CD27 molecule	Transmembrane receptor
CMKLR1	Chemokine-like receptor 1	G-protein coupled receptor
CSF2RA	Colony stimulating factor 2 receptor, alpha	Transmembrane receptor
CXCL10	Chemokine(C-X-C motif) ligand 10	Cytokine
CXCL12	Chemokine(C-X-C motif) ligand 12	Cytokine
CXCR3	Chemokine(C-X-C motif)receptor 3	G-protein coupled receptor
CXCR4	Chemokine(C-X-C motif)receptor 4	G-protein coupled receptor
CXCR5	Chemokine(C-X-C motif)receptor 5	G-protein coupled receptor
ERBB2	V-erb-b2 erythroblastic leukemia viral oncogene homolog 2	Kinase
FADD	Fas (TNFRSF6)-associated via death domain	Other
FGF23	Fibroblast growth factor 23	Growth factor
FGFRL1	Fibroblast growth factor receptor-like 1	Transmembrane receptor
HGF	Hepatocyte growth factor (hepapoietin A; scatter factor)	Growth factor
HRG	Histidine-rich glycoprotein	Other
IFNB1	Interferon, beta 1, fibroblast	Cytokine
IFNG	Interferon, gamma	Cytokine
IL12B	Interleukin 12B	Cytokine
IL15RA	Interleukin 15 receptor, alpha	Transmembrane receptor
IL16	Interleukin 16	Cytokine
IL18BP	Interleukin 18 binding protein	Other
IL1β	Interleukin 1, beta	Cytokine
IL20RA	Interleukin 20 receptor, alpha	Transmembrane receptor
IL22RA2	Interleukin 22 receptor, alpha 2	Transmembrane receptor
IL25	Interleukin 25	Cytokine
IL27	Interleukin 27	Cytokine
IL29	Interleukin29(interferon, lambda 1)	Other
IL2RA	Interleukin 2 receptor, alpha	Transmembrane receptor
IL32	Interleukin 32	Other
IL6	Interleukin 6 (interferon, beta 2)	Cytokine
IL6R	Interleukin 6 receptor	Transmembrane receptor
IL7	Interleukin 7	Cytokine
KIT	V-kit Hardy-Zuckerman 4 feline sarcoma viral oncogene homolog	Kinase
LTA	Lymphotoxin alpha (TNF superfamily, member 1)	Cytokine
LTBP1	Latent transforming growth factor beta binding protein 1	Other
MET	Met proto-oncogene	Kinase
MMP19	Matrix metallopeptidase 19	Peptidase
NGF	Nerve growth factor	Growth factor
NOS2	Nitric oxide synthase 2, inducible	Enzyme
NRG2	Neuregulin 2	Growth factor
PECAM1	Platelet/endothelial cell adhesion molecule	Other
SAA1	Serum amyloid A1	Transporter
SELL	Selectin L	Other
SMAD9	SMAD family member 9	Transcription regulator
TGFB1	Transforming growth factor, beta 1	Growth factor
TIMP4	TIMP metallopeptidase inhibitor 4	Other
TLR3	Toll-like receptor 3	Transmembrane receptor
TMEFF2	Transmembrane with EGF-like and two follistatin-like domains 2	Other
TNF	Tumor necrosis factor	Cytokine
TNFSF10	Tumor necrosis factor (ligand) superfamily, member 10	Cytokine
TNFSF12	Tumor necrosis factor (ligand) superfamily, member 12	Cytokine
TNFSF14	Tumor necrosis factor (ligand) superfamily, member 14	Cytokine

The gene symbol, description and type corresponding to each protein are given.

Network construction based on these 59 proteins was performed using Ingenuity Pathway Analysis (IPA) software (Ingenuity Systems, http://www.ingenuity.com ). The official symbol of each protein was imported into the IPA software. Through IPA analysis, we identified a total of 7 networks based on functional connectivity. Three of these networks shared common proteins, and it was possible to generate a merged network (data not shown). The merged network is very complicated and includes a few proteins that are not on our protein list. We pruned the network by removing the proteins that were not on our list except for some common and important molecules, such as NFκB, IL12 (complex), p38 MAPK, JAK, STAT, IFN-α and IFN-β. In addition, we integrated two molecules, IAV and COX-2, into the merged network. IL32, IL29, IL27, IL1β and IFN-α/β/γ have been reported to inhibit viral replication [3,33-37]. Therefore, we obtained our initial IRN comprising 51 proteins (or complexes) and 198 interactions. The network is depicted in Additional file 1. The full name of each protein in the initial IRN is listed in Additional file 2.

### Simplifying the initial IRN based on conditional mutual information

PCA-CMI, which was originally proposed by Zhang et al. for inferring GRNs from gene expression data, considers the nonlinear dependence and topological structure of GRNs by employing a path consistency algorithm (PCA) based on conditional mutual information (CMI) [20]. In this study, we used the PCA-CMI method to distinguish direct (or causal) interactions from indirect associations.

For a discrete variable *X*, the entropy *H*(*X*) is the measure of average uncertainty of variable *X* and can be defined by:

(1) $$H\left(X\right)=-\sum_{x\in X}p\left(x\right)\log p\left(x\right),$$

where *p*(*x*) is the probability of each discrete value *x* in *X*.

Mutual information (MI) measures the dependency between two variables (genes or proteins). For discrete variables *X* and *Y*, MI is defined by the following equation:

(2) $$I\left(X,Y\right)=-\sum_{x\in X,y\in Y}p\left(x,y\right)\log\frac{p\left(x,y\right)}{p\left(x\right)p\left(y\right)}.$$

CMI measures conditional dependency between two variables given other variable(s). The CMI of variables *X* and *Y* given *Z* is defined as:

(3) $$I\left(X,Y|Z\right)=-\sum_{x\in X,y\in Y,z\in Z}p\left(x,y,z\right)\log\frac{p\left(x,y|z\right)}{p\left(x|z\right)p\left(y|z\right)}.$$

With the widely adopted Gaussian kernel probability density estimator, the equations (1), (2) and (3) can be easily calculated using the following equivalent equations [15,20].

(4) $$H\left(X\right)=\frac{1}{2}\log{\left(2\mathrm{\pi e}\right)}^{n}\left|C\right|,$$

(5) $$I\left(X,Y\right)=\frac{1}{2}\log\frac{\left|C\left(X\right)\right|\cdot \left|C\left(Y\right)\right|}{\left|C\left(X,Y\right)\right|},$$

(6) $$I\left(X,Y|Z\right)=\frac{1}{2}\log\frac{\left|C\left(X,Z\right)\right|\cdot \left|C\left(Y,Z\right)\right|}{\left|C\left(Z\right)\right|\cdot \left|C\left(X,Y,Z\right)\right|},$$

where *C* is the covariance matrix of variable *X*, |*C* | is the determinant of matrix *C*, and *n* is the number of variables in *C.*

A high MI value indicates that there is a close relationship between the variables, while a low MI value implies variable independence. Similarly, a high CMI indicates that there is a close relationship between the variables *X* and *Y* given variable *Z*, while a low CMI value represents independence between genes. If the MI or CMI value is smaller than a given threshold *θ*, the edge between the two proteins is deleted for the independence (See the detailed procedure of PCA-CMI in [20]).

**Figure 2:**
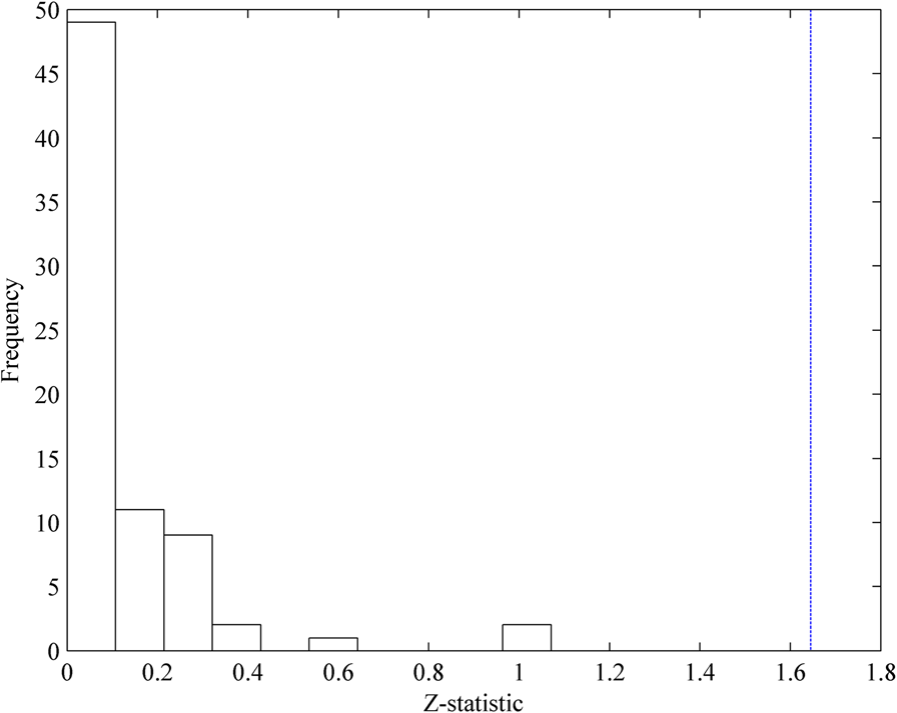
**The significance test for the selected threshold value of the MI and CMI.** The x-axis is the Z-statistic values of the deleted edges when the threshold value of MI and CMI is set to be 0.1. The y-axis represents the number of edges whose Z-statistic fall into the corresponding bins. The blue dashed line is the inverse cumulative distribution function of *N*(0,1) when using significant level α=0.1.

**Figure 3:**
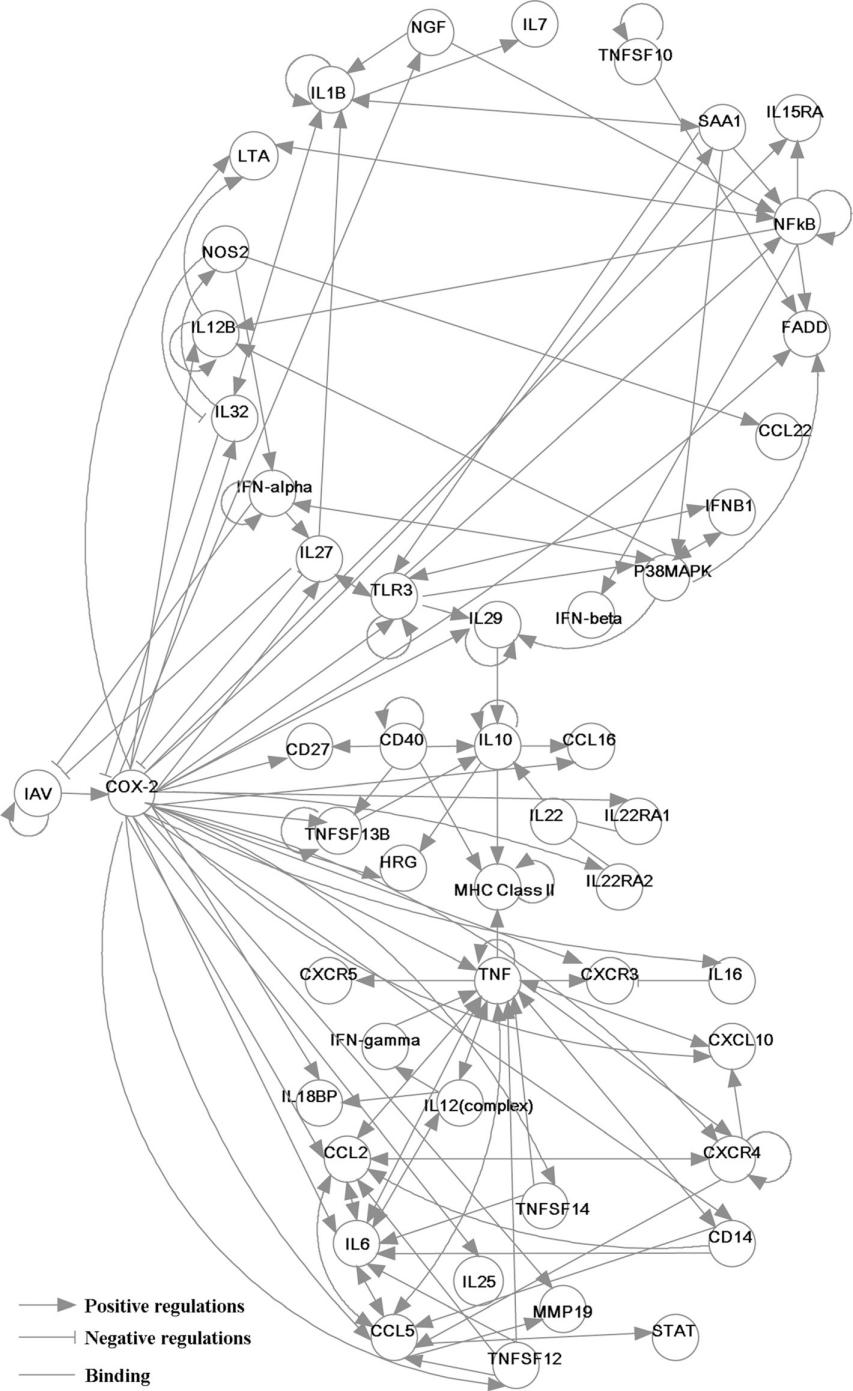
**The simplified inflammatory regulatory network based on mutual information.** The lines ended with arrows and bars denote positive and negative regulatory interactions, respectively. The lines without arrows or bars represent binding interactions.

We simplified the initial IRN based on PCA-CMI. We deleted the edges of the initial IRN with independent correlations recursively (i.e., from low to high order of independent correlation until no edge can be deleted). The threshold value *θ* of MI and CMI was set to 0.1, which was statistically tested by Z-statistic [15,20,38] (Figure 2). The simplified network, which contains 50 nodes and 142 directed edges, is depicted in Figure 3.

### Mathematical model of the network

To further obtain the more simplified IRN, we built nonlinear ordinary differential equations (ODEs) to model the reaction kinetics of the regulatory network. The ODEs describe the relationship between the reaction rate and the concentrations of the reactants. The change in concentration of a reactant is characterized by a function that takes the regulatory influence (activation or inhibition) of other reactants into account. The general form of nonlinear ODEs is described as follows:

$$\frac{dx_{i}}{\mathrm{dt}}=f_{i}\left(x_{1},x_{2},\ldots ,x_{m},k_{i\_j}\right)-d_{i}x_{i},$$

where *x*_*i*_ is the concentration of species *i*, *f*_*i*_ is a nonlinear function, *m* is the number of species in the system, *k*_*i_j*_ is the kinetic parameter with *i, j∈*{1, 2, … , *m*} and *d*_*i*_ is the degradation rate of species *i*.

Based on the law of mass action and Hill functions, the nonlinear ODEs including 50 equations and 192 kinetic parameters were built. All equations and their explanations and the initial concentrations of proteins are listed in Additional file 3.

### Estimation of the kinetic parameters in the model with the DE algorithm

The parameters in our ODEs can be classified into two categories of regulatory parameters: parameters representing activation or inhibition relations and degradation parameters representing the degradation of individual biomolecular species.

The problem that identifies the kinetic parameters in the model can be converted into the following nonlinear optimization problem, which is the minimization of the error between the simulation values in our model and the experimental data.

$$\underset{K}{\text{min}}\sum_{i=1}^{N}\sum_{j=1}^{M}\omega_{i}{\left(x_{i}{}^{\mathrm{sim}}\left(t_{j},K\right)-x_{i}{}^{\exp}\left(t_{j}\right)\right)}^{2},$$

where *x*_*i*_^exp^(*t*_*j*_) and *x*_*i*_^*sim*^(*t*_*j*_, *K*) are the experiment data and simulation value of the species *i* at *t*_*j*_ time point respectively. $\omega_{i}=1/{\left(\underset{j}{\text{max}}\;x_{i}{}^{\exp}\left(t_{j}\right)\right)}^{2}$, *K* is the parameter set consisting of all the parameters in the model, *N* is the number of species and *M* is the number of time points in the biological experiments.

A wide variety of global optimization techniques have been developed to address nonlinear optimization problems [39-42]. The DE algorithm, proposed by Storn and Price [42], is a very successful and powerful population-based stochastic search technique for solving global optimization problems and has been widely applied in many scientific and engineering fields [43,44]. The DE algorithm is described as follows:

Step 1. Initialization: Generate random initial population *X*_*G*_={ *X*_*1,G*_ , *X*_*2,G*_ ,…, *X*_*N,G*_ }, where *X*_*i,G*_={ *X*_*i1*_*,*_*G*_ , *X*_*i2*_*,*_*G*_ ,…, *X*_*iD*_ *,*_*G*_ }. *N* is the population size , G is the generation, *X*_*i,G*_ is a rate constant set and *D* is the amount of the kinetic parameters.

Step 2. Genetic operation:

$$U_{\mathrm{ji},G+1}=\left\{\begin{array}{l} V_{\mathrm{ji},G+1},\mathrm{if}\;\left(\mathrm{rand}\left(b\left(j\right)\right)\leq \mathrm{CR}\right)\;\mathrm{or}\;j=\mathrm{rnbr}\left(i\right)\\ X_{\mathrm{ji},G+1},\mathrm{if}\;\left(\mathrm{rand}\left(b\left(j\right)\right)>\mathrm{CR}\right)\;\mathrm{or}\;j\neq \mathrm{rnbr}\left(i\right) \end{array}\right),j=1,2,\cdots ,D,$$

where *rand*(*b*(*j*)) is the *j*th evaluation of a uniform random number in [0,1], *CR* is the crossover constant in [0,1] and *rnbr*(*i*) is a random indexes in {1,…,*D*}.

1) Mutation: *V*_*i,G+1*_=*X*_*r1,G*_+*F*(*X*_*r2,G*_- *X*_*r3,G*_), *r*_*1*_*, r*_*2*_*, r*_*3*_∈{1,…,*N*}\{i}, *F*∈ [0,2].

2) Crossover: *U*_*i,G+1*_=*(U*_*1i,G+1*_,…*,U*_*Di,G+1*_)

Step 3. Selection: If *f*(*U*_*i*,*G*_) ≤ *f*(*X*_*i*,*G*_), then *X*_*i*,*G* + 1_ = *U*_*i*,*G*_, else *X*_*i*,*G* + 1_ = *X*_*i*,*G*_, where *f* is the objection function.

### Average relative error

The average relative error (ARE) is defined as follows.

$$\mathrm{ARE}=\frac{1}{n}\sum_{i=1}^{n}\frac{\left|Y^{\mathrm{sim}}\left(t_{i}\right)-Y^{\exp}\left(t_{i}\right)\right|}{Y^{\exp}\left(t_{i}\right)},$$

where *Y*^sim^(*t*_*i*_) and *Y*^exp^(*t*_*i*_) are the simulation and experiment values of the protein at time point *t*_*i*_ and *n* is the number of samples. In this study, *n*=6.

### Sensitivity analysis

Sensitivity analysis is a useful way to investigate the effects of parameters variations on changes in the model outputs. We formulate the sensitivity *s*_*i*_(*t*) of parameter *P* at time *t* as follows:

$$s_{i}\left(t\right)=\frac{\partial O_{i}\left(t\right)}{O_{i}\left(t\right)}/\frac{\partial P\left(t\right)}{P\left(t\right)}\approx \left(|O_{i}\left(P+\mathrm{\Delta P},t\right)-O_{i}\left(P,t\right)|/O_{i}\left(P,t\right)\right)/\left(\mathrm{\Delta P}/P\right),$$

where *O*_*i*_(t) is the *i*-th model output at time *t*, *P* is the parameter, Δ*P* is a small perturbation of *P*.

Then we define the sensitivity *S*_*i*_ of the *i*-th model output with respect to parameter *P* blow.

$$S_{i}=\frac{1}{n}\sum_{t=1}^{n}s_{i}\left(t\right),$$

where *n* is the number of samples (time points).

### Enrichment analysis

We conducted a functional enrichment analysis for the network based on GO Biological Processes (BP) terms and the KEGG pathway with the DAVID bioinformatics database [45]. The enrichment significance was determined by the DAVID tool. The P-values were then corrected for the false discovery rate (FDR). In this study, all the proteins other than IAV in the network are mapped with the DAVID database. For the complex, one member of the complex was mapped. The criterion for statistically significant enrichment was an FDR adjusted p-value less than 0.002.

## Results

### The optimized IRN based on the experimental data

The initial and simplified IRNs (Figure 3) were constructed using IPA software and the PCA-CMI algorithm (See the Methods), respectively. To further optimize the network according to the experimental data, we first estimated all parameters in our nonlinear ODEs by the DE algorithm (See the Methods). The DE algorithm was carried out ten times, and the best parameter set was obtained, which is listed at Additional file 4: Table S2.

**Figure 4:**
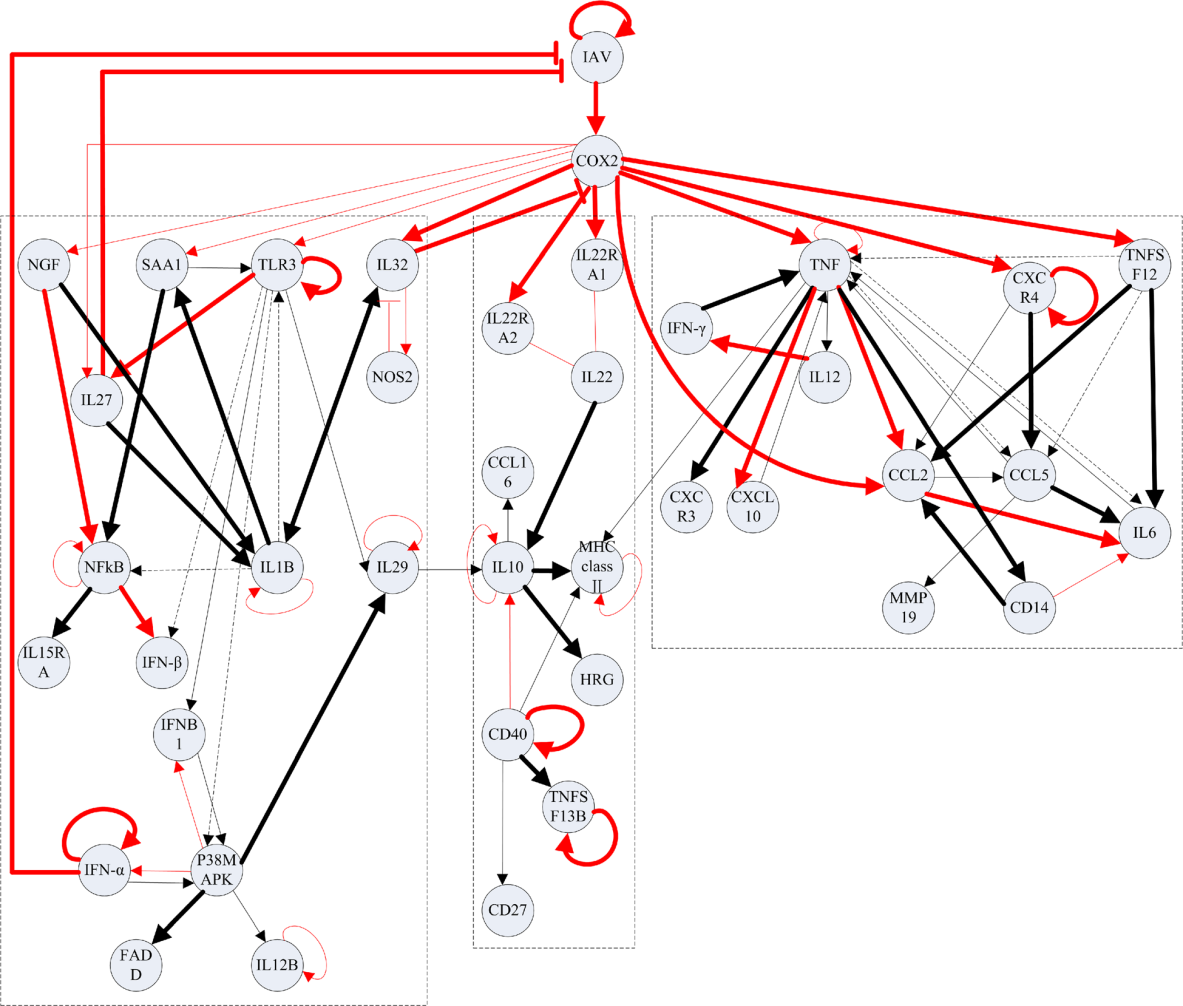
**The optimized inflammatory regulatory network.** The lines ended with arrows and bars denote positive and negative regulatory interactions, respectively.The lines without arrows or bars represent binding interactions. The dashed lines indicated the false positive interactions identified by the proposed method. The red and black lines stand for the regulatory interactions which have been validated by biological experiments and are predicted by the proposed method, respectively. The significant and non-significant regulations are denoted by thick and thin lines, respectively.

Second, we further deleted some nodes and edges to simplify the IRN according to the following rules. If the optimal value of the kinetic parameter *k*_*i*_*j*_ was zero, we deleted the directed edge, which indicates that biomolecular *j* does not regulate biomolecular *i* in the network. Furthermore, if there was no edge to connect with biomolecular *i*, we deleted the node *i* in the network. Finally, if the node *i* has been deleted in the network, the degradation rate *d*_*i*_ was set to zero in the numerical simulation. The optimized IRN is shown in Figure 4.

**Figure 5:**
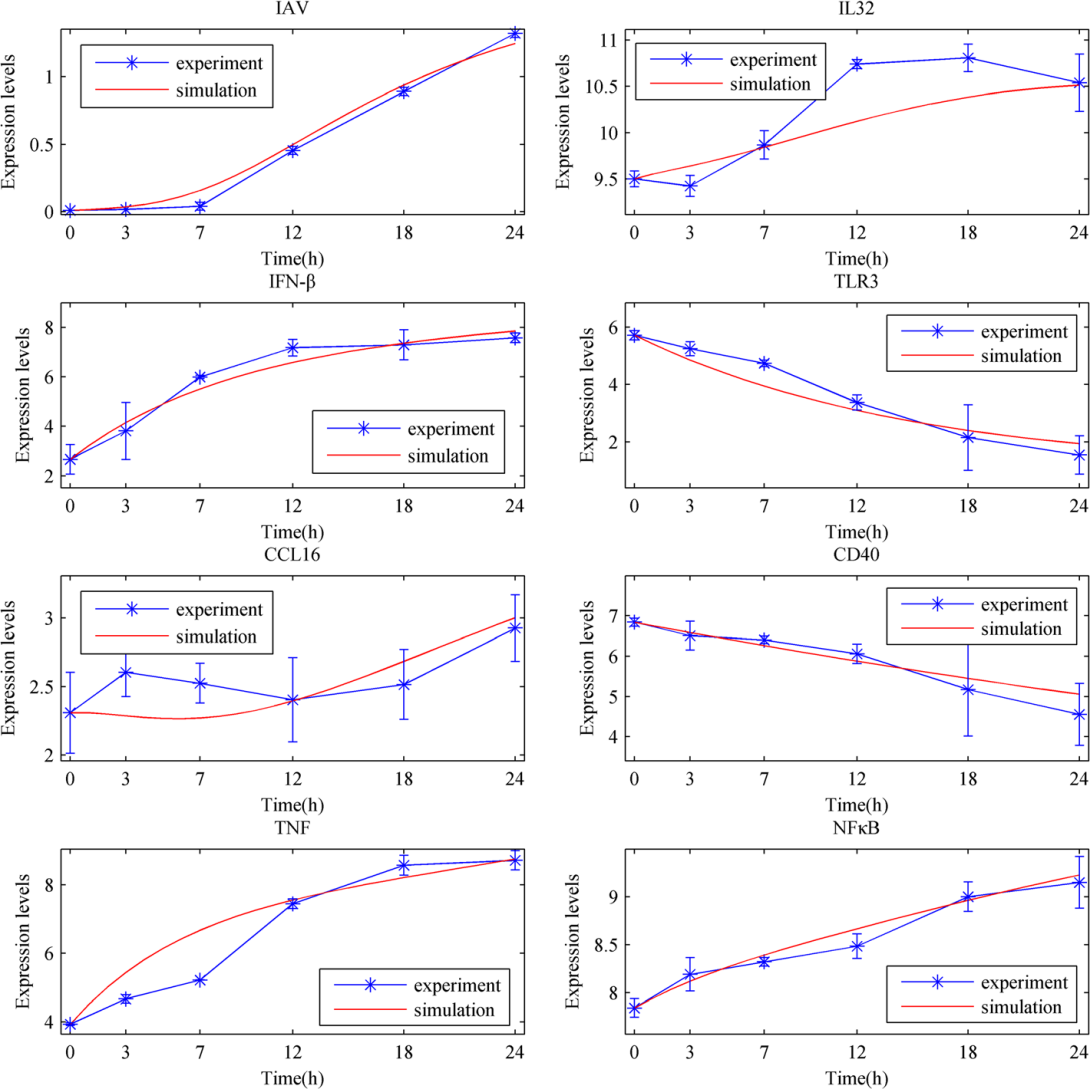
**Comparisons between the numerical simulation results and experimental data of IAV, IL32, IFN-β, TLR3, CCL16, CD40, TNF, NFκB.** The blue and red lines denote the experiment and simulation results, respectively. The stars represent experimental data at each time point. The experimental errors are also plotted as short bars at each time point.

**Figure 6:**
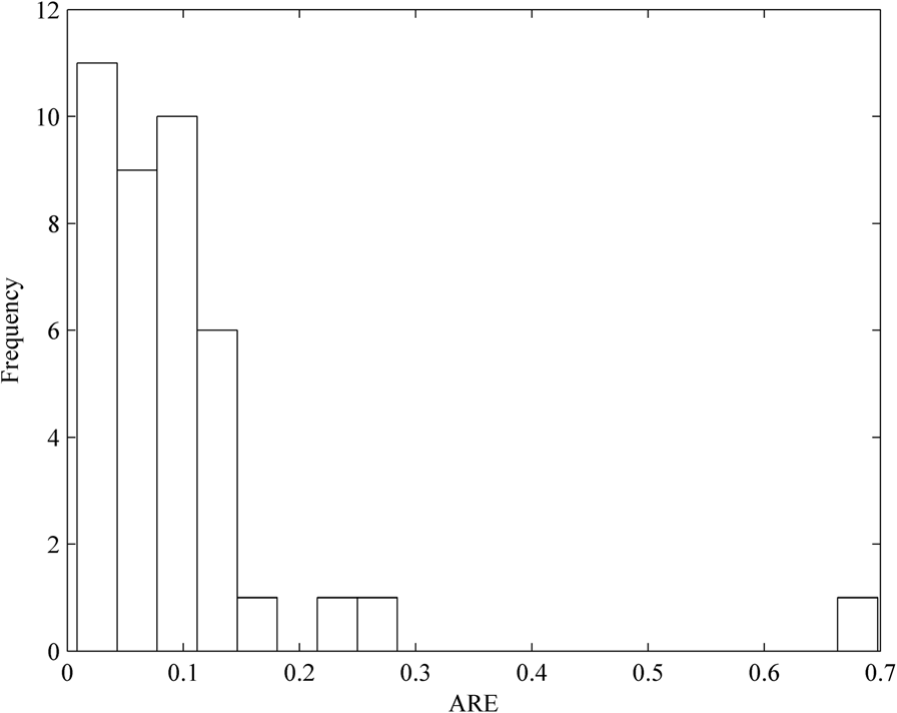
**The distribution of the average relative errors (AREs) for the numerical simulation of the proteins in the optimized network.** The y-axis represents the number of proteins whose AREs fall into the corresponding bins.

**Figure 7:**
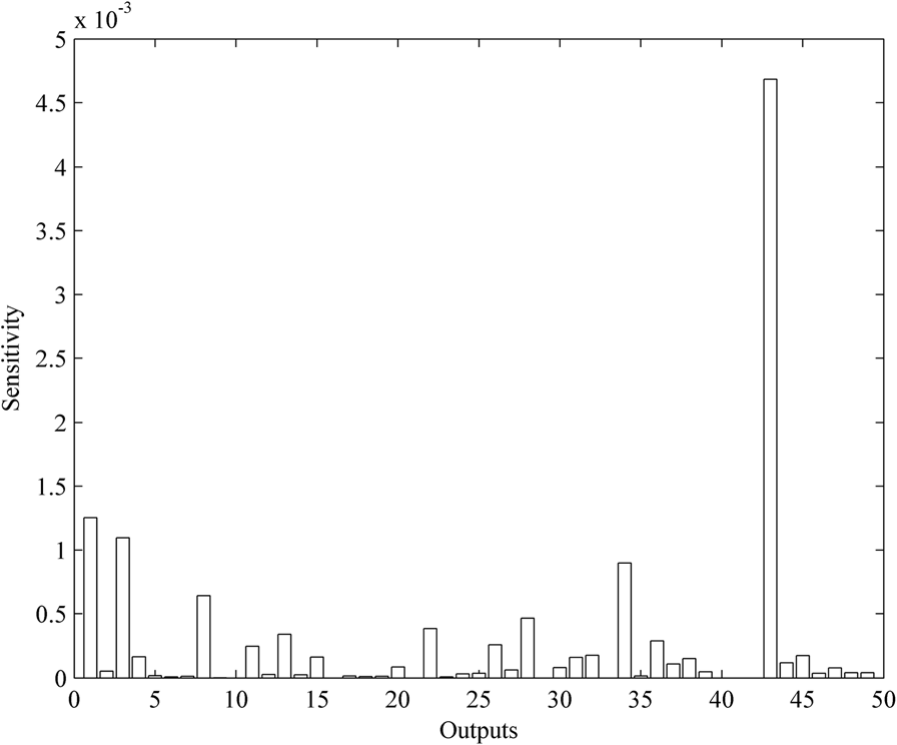
**The sensitivity analysis for the parameters in the ODE models.** The x-axis is the outputs (proteins) in the model and the y-axis is the calculated sensitivity.

Based on the optimal parameters, we performed a numerical simulation for all nodes in the network for comparison with the experimental data. The dynamical processes of 8 key proteins are plotted in Figure 5 and those of other proteins are displayed in Additional file 5. The average relative errors (AREs) of the 98% proteins are less than 0.3, and those of the 2% proteins are within the interval [0.3, 0.7] (Figure 6). These results indicated the fidelity of the obtained IRN. In addition, from the dynamical viewpoint, sensitivity analysis of the ODE models is very important to quantify the reliability of the parameters (regulatory strength between two genes) in the model (See the Methods). The results of the sensitivity analysis showed that the concentrations of the proteins are not sensitive to the perturbation of parameters (Figure 7), which indicating the reliability of the obtained IRN.

### Prediction of regulatory interactions in IRN

Among the regulatory interactions in the optimized network, 45 interactions have been reported in the literature and are represented by red lines in Figure 4. In addition, 37 new regulatory interactions have been predicted from the network and are denoted by black lines in Figure 4. Furthermore, the statistical significance of these regulations between paired proteins was tested using the method presented in the literature [15,20]. The significant and non-significant regulations were denoted by thick and thin lines in Figure 4, respectively. The number of significant and non-significant regulations was summarized in Table 2. The results demonstrated that most of the predicted regulatory interactions, which are the same as the validated experimental interactions, are statistically significant.

**Table 2:** The number of significant and non-significant regulations

	**Validated**	**Predicted**
Significant	24	21
Non-significant	21	16

The second and third columns are the number of regulations validated by biological experiments and predicted by our proposed method, respectively.

The presence of false positive interactions is a common problem in inferring a network. One source of false positive interactions is indirect effects (i.e., in a cascade A→ B→ C and A→ C, protein A regulates C and may be mediated by B, so the direct regulatory interaction A→ C may be a false positive interaction). Comparing the optimized IRN with the initial IRN, we have also identified 8 false positive interactions, which are shown by dashed lines in Figure 3. For example, the interactions involving the regulation of IFN-β by TLR3 and IL6 by TNF take effects during IAV infection through other chemical molecules. In our work, we have found that TLR3 regulates IFN-β through NFκB signaling, which is consistent with previous findings. The TLR3-induced NFκB signaling pathway is triggered by the virus, and NFκB regulates expression of the proinflammatory molecule IFN-β in the immune responses [46]. We have also found that TNF regulates IL6 mediated by the activation of CCL2 or CD14. The interactions in the optimized network are further classified, and detailed descriptions are presented in Additional file 6: Table S3.

### Identification of the important biological processes and pathways

To gain further insight into the biological interpretation of the optimized IRN during IAV infection, we have performed Biological Process (BP) terms and a KEGG pathway enrichment analysis of all the species other than IAV in the optimized IRN (See the Methods). Functional enrichment analysis of the species was conducted using DAVID [45]. The annotation analysis shows enrichment in BP involved in the defense response, inflammatory response, immune response and regulation of cytokine production. The top 10 enriched BP are listed in Table 3, and all detailed lists of the significantly enriched BP terms (FDR<0.002) are available in Additional file 7. In addition, network ontology analysis (NOA) which annotates biological networks [47], was used to analyze the enriched functions of the optimized IRN. The results of NOA are shown in Additional file 7. The functions of the optimized IAV-induced IRN are enriched in the response to stimulus, immune system process, inflammatory response, response to wounding and positive regulation of cellular processes, which are similar to the results by employing DAVID. The functional annotations of the optimized IRN reveal that the reconstructed IRN functions reasonably well and they reflect the defense response, immune response, response to wounding and regulation of cytokine production are the important processes of IAV-induced inflammatory response.

**Table 3:** Top 10 significantly enriched GO terms

**GO term (Biological Processes)**	**Count**	**FDR**
GO:0006952~defense response	24	7.01e-20
GO:0006954~inflammatory response	18	9.38e-16
GO:0009617~response to bacterium	15	2.53e-14
GO:0006955~immune response	21	2.65e-14
GO:0009611~response to wounding	18	3.56e-12
GO:0006935~chemotaxis	12	2.35e-10
GO:0042330~taxis	12	2.35e-10
GO:0002684~positive regulation of immune system process	13	6.24e-10
GO:0051240~positive regulation of multicellular organismal process	13	8.39e-10
GO:0001817~regulation of cytokine production	12	9.18e-10

The second column is the number of proteins from our protein list in the corresponding GO term.

The KEGG pathway enrichment analysis have identified a total of 8 pathways significantly enriched in this network (FDR<0.002). These pathways are shown in Table 4. Among them, the Toll-like receptors, the RIG-I-like receptors and the NOD-like receptors have been shown to be the main pattern-recognition receptors (PRR) by which the innate immune system recognizes the influenza virus infection [48]. Moreover, the NOD-like receptors play a primary role in host defence against invading pathogens and regulating NFκB signalling, IL1β production, and cell death, indicating that they are crucial to the pathogenesis of a variety of inflammatory human diseases [49]. The cytokine-cytokine receptor interaction and Jak-STAT signalling pathway are also well known antiviral response pathways [50,51].

**Table 4:** Significantly enriched KEGG pathways

**KEGG pathway term**	**Count**	**FDR**	**References**
Hsa04060:Cytokine-cytokine receptor interaction	23	2.49e-19	[49,50]
Hsa04620:Toll-like receptor signaling pathway	14	5.11e-12	[47,50,52]
Hsa04672:Intestinal immune network for IgA production	8	1.07e-05	Predicted
Hsa04630:Jak-STAT signaling pathway	11	1.30e-05	[36,49,50]
Hsa04622:RIG-I-like receptor signaling pathway	8	1.51e-04	[47,53-55]
Hsa04623:Cytosolic DNA-sensing pathway	7	7.10e-04	Predicted
Hsa04621:NOD-like receptor signaling pathway	7	0.001461	[47,48,50]
Hsa05330:Allograft rejection	6	0.001944	Predicted

The second column is the number of proteins from our protein list in the corresponding GO term. In the last column, the corresponding references of the pathways involved in influenza A virus infection are given, and the terms ”predicted” indicate that the pathways have not been reported by previous literatures.

Three additional identified pathways have not been demonstrated to be associated with IAV infection. The intestinal immune network for IgA production significantly enriched (FDR=1.07e-05). Some researchers have reported that serum IgA is an inflammatory antibody that interacts with FcαRI on effector immune cells and may function as a second line of defence by eliminating pathogens that have breached the mucosal surface [56,57]. The detection of cytosolic DNA is related to the induction of IFN-α/β and other pro-inflammatory cytokines [58-61]. Cytosolic DNA has also been shown to activate the TBK1, IRF3 and the caspase-1-dependent maturation of IL-1β and IL-18 [58,62]. Allograft rejection is also enriched significantly. Some authors have reported that influenza infections are associated with allograft rejection, but there is no evidence that IAV triggers the acute allograft rejection episodes [63-65]. In our results, under the stimulation of IAV, the allograft rejection pathway is significantly enriched. These three pathways lack literature support but may be promising novel pathways and need the experimental validation.

## Discussion

The induction of pro-inflammatory cytokines such as COX-2, TNF, IFNs, IL27 and CXCL10 is essential for the host immune response during virus infection, but inappropriately sustained induction causes cytokine-storms, which are associated with a wide variety of infectious diseases [66,67]. Because of the complexity of the inflammatory response, it is necessary to study the underlying mechanisms of inflammatory response based on a network approach. In this study, we proposed a nonlinear ODE-model based computational method to construct a cell-specific IRN during IAV infection. The main contributions of this study include three aspects. First, we built the large-scaled nonlinear ODE model of the network including 50 equations and 192 kinetic parameters. Most of model-based studies for inferring networks are based on linear ODE models or discrete models [14-16], and these linear ODEs are approximated by difference equations or the steady-state assumption, which are easily solved by classical optimization algorithms or software. However, the regulatory interactions in real biological networks are often non-linear. Therefore, the non-linear ODE model can better describe the complicated regulatory networks. The comparison study for the advantage of involving nonlinear items in the model was also performed by using linear ODE model to describe the regulatory network. The AREs in the linear model exhibited significantly higher values than those in the nonlinear model (Additional file 8: Figure S1, P-value<0.001). These results indicated that the non-linear ODE model can better describe the complicated regulatory networks. Second, we combined the DE algorithm with a priori knowledge to refine the nonlinear ODEs and solve the nonlinear optimization problem derived from constructing the network. This nonlinear optimization problem is difficult to solve using classical optimization algorithms because of high nonlinearity and no explicit expression. Although DE algorithm is a published stochastic search technique, it is a repeated process from the model to optimization and then from improved model to optimization. If the model is not correct, the best optimization algorithm is also useless. Our nonlinear ODE model has been repeatedly adjusted. Finally, global errors that reflect the effectiveness of fitting the reconstructed network to experimental data are presented. In most studies based on the linear model systems, they did not provide the errors or only gave the residual errors (local errors) that cannot quantify the real error between the networks and the experimental data.

Because our proposed method integrated gene expression data with a priori knowledge of topological structure from literature and IPA software, it cannot compare with the published purely data-driven methods to evaluate the predictive results. However, these published excellent works may help us to find a more appropriate way to evaluate the approaches that combined the experimental data and a priori knowledge in the future.

An increasing number of researchers have focused on the gene expression profile of host cells infected by influenza virus [68-70]. However, most reports involve a single gene or pathway [52,53,71]. Few studies have focused on the systematic analysis of the regulation of the cell-signaling cascade by IAV. To understand the global regulatory mechanisms of the inflammatory response during IAV infection, we conducted a pathway enrichment analysis of the optimal IRN with the KEGG database. From our results, a few host cellular signaling pathways stimulated by IAV infection have been identified. Some of these signaling pathways are critical to the innate immune response of the host cell against influenza virus, such as the Toll-like receptor, the RIG-I-like receptor and the NOD-like receptor pathways [48,54]. The activation of the TLR signaling pathway results in the stimulation of both innate and adaptive immune responses, and TLR agonists may represent an effective and broad-spectrum antiviral strategy to combat influenza viruses [71]. Several virus-encoded components that antagonize RLR signalling interact with and inhibit the IFN-α/β activation pathway using both RNA-dependent and RNA-independent mechanisms [55,72].

Among the three novel pathways identified in our study, the functions of IgA have been studied [56,57]. Secretory immunoglobulin A (SIgA) is the major antibody isotype present in mucosal secretions and has many functional attributes, both direct and indirect, serving to prevent infective agents such as bacteria and viruses from breaching the mucosal barrier [42]. Many DNA-sensors have been reported, such as IFI16, RNA Polymerase III, DAI, AIM2, NLRP3, LRRFIP1 and DDX9/36. They play an important role in IFN-α/β and cytokine production [54,58,73]. For example, IFI16 can induce the inflammasome in response to Kaposi’s sarcoma-associated herpesvirus infection and act as a mediator of the anti-inflammatory actions of type I IFNs [73-76]. AIM2 triggers the assembly of the inflammasome, culminating in caspase-1 activation, IL-1β maturation and pyroptotic cell death [77]. LRRFIP1 has been shown to contribute to the production of IFN-β induced by vesicular stomatitis virus (VSV) and Listeria monocytogenes in macrophages [78]. This evidence indicates that DNA sensors play an important role in virus infection. However, these results need further biological experimental verification.

It should be noted that the protein activity profiles are substituted with the corresponding gene expression levels in the computation in the study because the protein activity profile data have not been easily obtained thus far. Therefore, there may be some discrepancy when modelling the network. In addition, the network we constructed does not involve RNA components, such as target mRNAs, micro-RNAs (mi-RNAs) or other non-coding RNAs, which may also modulate signals at many steps. Recent studies have provided evidence of a potential role for long non-coding RNAs (lncRNAs) in regulating inflammatory gene expression [79,80]. Emerging evidence shows that mi-RNAs have been clearly implicated in the regulation of inflammatory responses [81-85]. To better understand the molecular mechanisms of the inflammatory response during IAV infection, it requires the challenging process of constructing inflammatory regulatory networks by integrating different types of data, such as gene expression data, protein activity profiles, mi-RNAs expression profiles and Chip-seq data.

## Conclusions

A cell-specific IRN in IAV infection has been constructed based on the proposed method. Furthermore, 37 new regulatory interactions were predicted and 8 false positive interactions of IRN and 3 novel pathways were identified in the study. These new findings provide insight into our understanding of the mechanism of inflammatory response in IAV infection. Understanding the pivotal role of signaling pathways during IAV infection may provide new insight into therapeutic strategies for the control of virus infection and inflammatory response. Our findings also have significant implications on the development of biomarkers for infectious disease.

## Abbreviations

BP: Biological processes; CMI: Conditional mutual information; COX-2: Cyclooxygenase-2; DE: Differential evolution; FDR: False discovery rate; GEO: Gene expression omnibus; GO: Gene ontology; GRN: Gene regulatory network; IAV: Influenza A virus; IPA: Ingenuity pathway analysis; IRN: Inflammatory regulatory network; KEGG: Kyoto encyclopedia of genes and genomes; MI: Mutual information; mi-RNAs: micro-RNAs; ODE: Ordinary differential equation; PCA: Path consistency algorithm; PRR: Pattern-recognition receptors; SIgA: Secretory immunoglobulin A.

## Competing interests

The authors have declared that no competing interests exist.

## Authors’ contributions

XFZ designed the study, SQJ analyzed the data and performed the simulations, SQJ and XFZ wrote the paper. All authors read and approved the final manuscript.

## Supplementary Material

Additional file 1 This file includes the initial inflammatory regulatory network by using Ingenuity Pathway Analysis (IPA) software based on the data from Gene Expression Omnibus (GEO) database under the GEO accession number GSE28166 and the website for all uploaded source codes and data. Click here for file

Additional file 2 This file includes the full name of each protein in the initial IRN. Click here for file

Additional file 3 This file includes the mathematical model according to the simplified network and additional Table S1 list the initial values of all components in the model. Click here for file

Additional file 4 This file includes one additional Table S2 that lists the optimal parameter values obtained by DE algorithm. Click here for file

Additional file 5 This file includes the comparisons between the numerical simulation results and experimental data of proteins in the optimized network. Click here for file

Additional file 6 This file includes one additional Table S3 that lists the classification of the interactions in the optimized network. Click here for file

Additional file 7 This file includes all significantly enriched BP terms by using DAVID and NOA. Click here for file

Additional file 8 This file includes the comparison study for the advantage of involving non-linear items in the model. Click here for file
