# Supplementary material for: Construction of the influenza A virus infection-induced cell-specific inflammatory regulatory network based on mutual information and optimization
Source: BMC Syst Biol. 2013 Oct 20;7:105. doi: 10.1186/1752-0509-7-105 (PMC4016583; doi:10.1186/1752-0509-7-105)
Supplement: Additional file 3 — This file includes the mathematical model according to the simplified network and additional Table S1 list the initial values of all components in the model. [file 1752-0509-7-105-S3.pdf]

This file includes the mathematical model according to the simplified network and additional Table S1 list the initial values of all components in the model.

(A) The built mathematical model according to the simplified network (Figure 3 in the main text)

$$\frac{dx_1}{dt} = \frac{k_1 x_1}{(0.5 + x_1)(1 + a_1 x_4)(1 + a_2 x_7)} - d_1 x_1 \quad \text{(S1)}$$

$$\frac{dx_2}{dt} = \frac{k_{2\_1} x_1}{(1 + a_3 x_4)(1 + a_4 x_5)} - d_2 x_2 \quad \text{(S2)}$$

$$\frac{dx_3}{dt} = k_3 x_3 + k_{3\_2} x_2 + k_{3\_15} x_{15} + k_{3\_47} x_{47} - d_3 x_3$$

$$\frac{dx_4}{dt} = k_{4\_2} x_2 + k_{4\_7} x_7 + k_{4\_15} x_{15} - d_4 x_4$$

$$\frac{dx_5}{dt} = k_{5\_2} x_2 + k_{5\_6} x_6 + \frac{k_{5\_14}}{1 + a_5 x_{14}} - d_5 x_5 \quad \text{(S3)}$$

$$\frac{dx_6}{dt} = k_6 x_6 + k_{6\_4} x_4 + k_{6\_5} x_5 + k_{6\_17} x_{17} + k_{6\_19} x_{19} - d_6 x_6$$

$$\frac{dx_7}{dt} = k_7 x_7 + k_{7\_14} x_{14} + k_{7\_47} x_{47} - d_7 x_7$$

$$\frac{dx_8}{dt} = k_{8\_48} x_{48} - d_8 x_8$$

$$\frac{dx_9}{dt} = k_{9\_46} x_{46} - d_9 x_9$$

$$\frac{dx_{10}}{dt} = k_{10\_6} x_6 - d_{10} x_{10}$$

$$\frac{dx_{11}}{dt} = k_{11} x_{11} + k_{11\_2} x_2 + k_{11\_47} x_{47} + k_{11\_48} x_{48} - d_{10} x_{10}$$

$$\frac{dx_{12}}{dt} = k_{12\_2} x_2 + k_{12\_48} x_{48} - d_{12} x_{12}$$

$$\frac{dx_{13}}{dt} = k_{13\_15} x_{15} + k_{13\_47} x_{47} - d_{13} x_{13}$$

$$\frac{dx_{14}}{dt} = k_{14\_5} x_5 - d_{14} x_{14}$$

$$\frac{dx_{15}}{dt} = k_{15} x_{15} + k_{15\_2} x_2 + k_{15\_4} x_4 + k_{15\_13} x_{13} + k_{15\_19} x_{19} - d_{15} x_{15}$$

$$\frac{dx_{16}}{dt} = k_{16\_2} x_2 + k_{16\_11} x_{11} + k_{16\_48} x_{48} - d_{16} x_{16}$$

$$\frac{dx_{17}}{dt} = k_{17\_2} x_2 - d_{17} x_{17}$$

$$\frac{dx_{18}}{dt} = k_{18} x_{18} - d_{18} x_{18}$$

$$\frac{dx_{19}}{dt} = k_{19\_2}x_2 + k_{19\_6}x_6 - d_{19}x_{19}$$

$$\frac{dx_{20}}{dt} = k_{20\_2}x_2 + k_{20\_18}x_{18} + k_{20\_47}x_{47} + k_{20\_48}x_{48} - d_{20}x_{20}$$

$$\frac{dx_{21}}{dt} = k_{21\_14}x_{14} - d_{21}x_{21}$$

$$\frac{dx_{22}}{dt} = k_{22}x_{22} + k_{22\_3}x_3 + k_{22\_25}x_{25} + k_{22\_26}x_{26} + k_{22\_43}x_{43} - d_{22}x_{22}$$

$$\frac{dx_{23}}{dt} = k_{23\_2}x_2 + k_{23\_22}x_{22} - d_{23}x_{23}$$

$$\frac{dx_{24}}{dt} = k_{24\_2}x_2 + k_{24\_25}x_{25} - d_{24}x_{24}$$

$$\frac{dx_{25}}{dt} = k_{25}x_{25} - d_{25}x_{25}$$

$$\frac{dx_{26}}{dt} = k_{26}x_{26} + k_{26\_2}x_2 + k_{26\_25}x_{25} - d_{26}x_{26}$$

$$\frac{dx_{27}}{dt} = k_{27\_2}x_2 + k_{27\_22}x_{22} - d_{27}x_{27}$$

$$\frac{dx_{28}}{dt} = k_{28}x_{28} + k_{28\_2}x_2 + k_{28\_9}x_9 + k_{28\_29}x_{29} + k_{28\_30}x_{30} + k_{28\_34}x_{34} + k_{28\_35}x_{35}$$
$$+ k_{28\_36}x_{36} + k_{28\_37}x_{37} + k_{28\_39}x_{39} + k_{28\_46}x_{46} - d_{28}x_{28}$$

$$\frac{dx_{29}}{dt} = k_{29\_2}x_2 - d_{29}x_{29}$$

$$\frac{dx_{30}}{dt} = k_{30\_2}x_2 - d_{30}x_{30}$$

$$\frac{dx_{31}}{dt} = k_{31\_2}x_2 + k_{31\_28}x_{28} + \frac{k_{31\_40}}{1 + a_6x_{40}} - d_{31}x_{31} \qquad \text{(S4)}$$

$$\frac{dx_{32}}{dt} = k_{32}x_{32} + k_{32\_2}x_2 + k_{32\_28}x_{28} - d_{32}x_{32}$$

$$\frac{dx_{33}}{dt} = k_{33\_28}x_{28} - d_{33}x_{33}$$

$$\frac{dx_{34}}{dt} = k_{34\_2}x_2 + k_{34\_28}x_{28} + k_{34\_32}x_{32} - d_{34}x_{34}$$

$$\frac{dx_{35}}{dt} = k_{35\_2}x_2 + k_{35\_28}x_{28} + k_{35\_30}x_{30} + k_{35\_32}x_{32} + k_{35\_36}x_{36} + k_{35\_37}x_{37} + k_{35\_39}x_{39} - d_{35}x_{35}$$

$$\frac{dx_{36}}{dt} = k_{36\_2}x_2 + k_{36\_28}x_{28} + k_{36\_30}x_{30} + k_{36\_32}x_{32} + k_{36\_35}x_{35} + k_{36\_37}x_{37} + k_{36\_39}x_{39} - d_{36}x_{36}$$

$$\frac{dx_{37}}{dt} = k_{37\_2}x_2 + k_{37\_28}x_{28} - d_{37}x_{37}$$

$$\frac{dx_{38}}{dt} = k_{38\_2}x_2 + k_{38\_36}x_{36} - d_{38}x_{38}$$

$$\frac{dx_{39}}{dt} = k_{39\_2}x_2 + k_{39\_28}x_{28} + k_{39\_29}x_{29} + k_{39\_30}x_{30} + k_{39\_35}x_{35} + k_{39\_36}x_{36} + k_{39\_37}x_{37} + k_{39\_46}x_{46} - d_{39}x_{39}$$

$$\frac{dx_{40}}{dt} = k_{40\_2}x_2 - d_{40}x_{40}$$

$$\frac{dx_{41}}{dt} = k_{41\_2}x_2 - d_{41}x_{41}$$

$$\frac{dx_{42}}{dt} = k_{42\_2}x_2 + k_{42\_46}x_{46} - d_{42}x_{42}$$

$$\frac{dx_{43}}{dt} = k_{43\_44\_45}x_{44}x_{45} - d_{43}x_{43} \qquad \text{(S5)}$$

$$\frac{dx_{44}}{dt} = k_{44\_2}x_2 - k_{43\_44\_45}x_{44}x_{45} - d_{44}x_{44} \qquad \text{(S6)}$$

$$\frac{dx_{45}}{dt} = k_{45\_2}x_2 - k_{43\_44\_45}x_{44}x_{45} - d_{45}x_{45} \qquad \text{(S7)}$$

$$\frac{dx_{46}}{dt} = k_{46\_28}x_{28} + k_{46\_39}x_{39} - d_{46}x_{46}$$

$$\frac{dx_{47}}{dt} = k_{47\_7}x_7 + k_{47\_13}x_{13} + k_{47\_15}x_{15} + k_{47\_19}x_{19} - d_{47}x_{47}$$

$$\frac{dx_{48}}{dt} = k_{48}x_{48} + k_{48\_15}x_{15} + k_{48\_16}x_{16} + k_{48\_17}x_{17} + k_{48\_19}x_{19} - d_{48}x_{48}$$

$$\frac{dx_{49}}{dt} = k_{49}x_{49} + k_{49\_22}x_{22} + k_{49\_25}x_{25} + k_{49\_28}x_{28} - d_{49}x_{49}$$

$$\frac{dx_{50}}{dt} = k_{50\_36}x_{36} - d_{50}x_{50}$$

We gave the following explanations for the above equations.

The concentration of component X_i that changes over time can be described by an ordinary differential equation (ODE)

$$\frac{dX_i}{dt} = v_{i,production} - v_{i,\deg radation}$$

where $v_{i,production}$ and $v_{i,\deg radation}$ represent the production rate and the degradation rate of reactant X_i, respectively. In general, based on the law of mass action, we assume that the production rates and the degradation rates of these components are linearly proportional to their concentrations. But several special processes (S1-S7) are different based on the available experimental data and network topology.

1. The regulations of the positive and negative feedbacks are described using Hill functions.

(1) In Eq.S1, we used the term $\frac{k_1 x_1}{0.5 + x_1}$ to represent the replication of x_1 (influenza A virus, IAV),

which is equivalent to the positive feedback, the terms $\frac{1}{1+a_1x_4}$ and $\frac{1}{1+a_2x_7}$ to represent the negative feedbacks of x_4 (IL27) and x_7 (IFN-alpha) to x_1 (IAV), respectively.

$$\frac{dx_1}{dt}=\frac{k_1x_1}{(0.5+x_1)(1+a_1x_4)(1+a_2x_7)}-d_1x_1 \qquad \text{(S1)}$$

(2) In Eq.S2, x_4 (IL27) and x_5 (IL32) have also a negative feedback to x_2 (COX-2), respectively.

$$\frac{dx_2}{dt}=\frac{k_{2\_1}x_1}{(1+a_3x_4)(1+a_4x_5)}-d_2x_2 \qquad \text{(S2)}$$

(3) In Eqs.S3, x14(NOS2) has a negative feedback to x5 (IL32)

$$\frac{dx_5}{dt}=k_{5\_2}x_2+k_{5\_6}x_6+\frac{k_{5\_14}}{1+a_5x_{14}}-d_5x_5 \qquad \text{(S3)}$$

(5) In Eqs.S4, x40 (IL16) has a negative feedback to x31 (CXCR3)

$$\frac{dx_{31}}{dt}=k_{31\_2}x_2+k_{31\_28}x_{28}+\frac{k_{31\_40}}{1+a_6x_{40}}-d_{31}x_{31} \qquad \text{(S4)}$$

2. Coordinate interactions are represented by multiplying together.

In Eq.S1, because x_1 (IAV) is coordinately regulated by interlinked positive and negative feedbacks, we use the multiply $\frac{k_1x_1}{0.5+x_1}\cdot\frac{1}{1+a_1x_4}\cdot\frac{1}{1+a_2x_7}$. Similarly, in Eq.S2, we use $\frac{k_{2\_1}x_1}{(1+a_3x_4)(1+a_4x_5)}$ to represent that the x_2 (COX-2) is regulated by two negative feedbacks altogether.

3. Binding interactions are formulated by linear product.

In Eqs. S5-S7, both x_{44} (IL22RA1) and x_{45} (IL22RA2) are the receptor factors of x_{43} (IL22) (See the additional file 2) and they altogether can bind to the x_{43} (IL22). Therefore, these terms are formulated by linear product.

(B) Table S1 The initial values of all components.

Chemical species	Initial value	Chemical species	Initial value	Chemical species	Initial value
x_1	0.01	x_{18}	9.69	x_{35}	10.15
x_2	7.29	x_{19}	8.99	x_{36}	3.82
x_3	3.22	x_{20}	12.16	x_{37}	5.79
x_4	9.05	x_{21}	1.61	x_{38}	2.42
x_5	9.50	x_{22}	2.65	x_{39}	9.78
x_6	7.20	x_{23}	2.31	x_{40}	1.26
x_7	8.62	x_{24}	3.81	x_{41}	2.86
x_8	2.66	x_{25}	6.84	x_{42}	4.54

x_9	3.25	x_{26}	3.51	x_{43}	0.31
x_{10}	4.45	x_{27}	5.53	x_{44}	5.35
x_{11}	1.69	x_{28}	3.94	x_{45}	2.73
x_{12}	9.30	x_{29}	4.08	x_{46}	2.63
x_{13}	2.66	x_{30}	8.34	x_{47}	8.26
x_{14}	2.24	x_{31}	4.40	x_{48}	7.84
x_{15}	5.72	x_{32}	7.84	x_{49}	5.86
x_{16}	3.17	x_{33}	3.72	x_{50}	6.92
x_{17}	3.08	x_{34}	1.53		

Abbreviations are as follows: *x*1: IAV (influenza A virus), *x*2: COX-2, *x*3: IL29, *x*4: IL27, *x*5: IL32, *x*6: IL1β, *x*7: IFN-alpha, *x*8: IFN-beta, *x*9: IFN-gamma, *x*10: IL7, *x*11: IL12B, *x*12: IL15RA, *x*13: IFNB1, *x*14: NOS2, *x*15: TLR3 , *x*16: LTA , *x*17: NGF, *x*18: TNFSF10, *x*19: SAA1, *x*20: FADD, *x*21: CCL22, *x*22: IL10 , *x*23: CCL16, *x*24: CD27, *x*25: CD40, *x*26: TNFSF13B, *x*27: HRG, *x*28: TNF, *x*29: TNFSF14, *x*30: TNFSF12, *x*31: CXCR3, *x*32: CXCR4, *x*33: CXCR5, *x*34: CXCL10, *x*35: CCL2, *x*36: CCL5, *x*37: CD14, *x*38: MMP19, *x*39: IL 6, *x*40: IL16, *x*41: IL25, *x*42: IL18BP, *x*43: IL22, *x*44: IL22RA1, *x*45: IL22RA2, *x*46: IL12, *x*47: P38MAPK, *x*48: NFκB, *x*49: MHC ClassⅡ, *x*50: STAT
