# Supplementary material for: Construction of the influenza A virus infection-induced cell-specific inflammatory regulatory network based on mutual information and optimization
Source: BMC Syst Biol. 2013 Oct 20;7:105. doi: 10.1186/1752-0509-7-105 (PMC4016583; doi:10.1186/1752-0509-7-105)
Supplement: Additional file 4 — This file includes one additional Table S2 that lists the optimal parameter values obtained by DE algorithm. [file 1752-0509-7-105-S4.pdf]

This file includes one additional Table S2 that lists the optimal parameter values obtained by DE algorithm.

Table S2 The optimal parameter values obtained by DE algorithm.

Kinetic parameter	Optimal value	Kinetic parameter	Optimal value	Kinetic parameter	Optimal value	Kinetic parameter	Optimal value
k_1	8.500e-05	k_{21_14}	0	k_{36_30}	0	d_3	4.500e-05
k_{2_1}	2.118e-05	k_{22}	1.427e-05	k_{36_32}	2.108e-05	d_4	8.800e-06
k_3	8.560e-06	k_{22_3}	1.000e-05	k_{36_35}	5.640e-06	d_5	4.792e-05
k_{3_2}	0	k_{22_25}	1.018e-06	k_{36_37}	0	d_6	9.215e-05
k_{3_15}	1.026e-05	k_{22_26}	0	k_{36_39}	0	d_7	7.200e-05
k_{3_47}	4.712e-05	k_{22_43}	3.984e-05	k_{37_2}	0	d_8	3.600e-05
k_{4_2}	8.000e-06	k_{23_2}	0	k_{37_28}	5.538e-05	d_9	1.802e-05
k_{4_7}	0	k_{23_22}	1.383e-05	k_{38_2}	0	d_{10}	0
k_{4_15}	2.821e-05	k_{24_2}	0	k_{38_36}	9.160e-06	d_{11}	2.672e-05
k_{5_2}	2.565e-05	k_{24_25}	1.000e-05	k_{39_2}	0	d_{12}	5.300e-05
k_{5_6}	3.942e-05	k_{25}	1.709e-05	k_{39_28}	0	d_{13}	3.000e-06
k_{5_14}	1.230e-06	k_{26}	6.925e-05	k_{39_29}	0	d_{14}	6.570e-06
k_6	8.300e-07	k_{26_2}	0	k_{39_30}	3.112e-05	d_{15}	4.592e-05
k_{6_4}	4.629e-05	k_{26_25}	1.800e-05	k_{39_35}	1.811e-05	d_{16}	0
k_{6_5}	2.050e-05	k_{27_2}	0	k_{39_36}	2.994e-05	d_{17}	2.683e-05
k_{6_17}	1.612e-05	k_{27_22}	7.500e-05	k_{39_37}	1.000e-05	d_{18}	0
k_{6_19}	0	k_{28}	7.200e-06	k_{39_46}	0	d_{19}	1.800e-05
k_7	6.354e-05	k_{28_2}	2.500e-05	k_{40_2}	0	d_{20}	4.194e-05
k_{7_14}	0	k_{28_9}	3.331e-05	k_{41_2}	0	d_{21}	0
k_{7_47}	1.000e-05	k_{28_29}	0	k_{42_2}	0	d_{22}	7.000e-05
k_{8_48}	3.268e-05	k_{28_30}	0	k_{42_46}	0	d_{23}	1.500e-05
k_{9_46}	2.200e-05	k_{28_34}	1.000e-07	$k_{43\_44\_45}$	1.000e-05	d_{24}	1.500e-05
k_{10_6}	0	k_{28_35}	0	k_{44_2}	2.710e-05	d_{25}	2.059e-05
k_{11}	1.128e-05	k_{28_36}	7.910e-06	k_{45_2}	3.000e-05	d_{26}	8.000e-05
k_{11_2}	0	k_{28_37}	0	k_{46_28}	8.950e-06	d_{27}	3.000e-05
k_{11_47}	7.910e-06	k_{28_39}	7.300e-07	k_{46_39}	0	d_{28}	4.700e-05
k_{11_48}	0	k_{28_46}	0	k_{47_7}	5.750e-06	d_{29}	0
k_{12_2}	0	k_{29_2}	0	k_{47_13}	1.412e-05	d_{30}	1.000e-05
k_{12_48}	6.000e-05	k_{30_2}	2.014e-05	k_{47_15}	0	d_{31}	6.000e-05
k_{13_15}	1.000e-06	k_{31_2}	0	k_{47_19}	0	d_{32}	4.563e-05
k_{13_47}	1.063e-05	k_{31_28}	5.000e-05	k_{48}	5.020e-06	d_{33}	0
k_{14_5}	1.000e-06	k_{31_40}	0	k_{48_15}	0	d_{34}	4.600e-05
k_{15}	2.700e-05	k_{32}	1.745e-05	k_{48_16}	0	d_{35}	8.300e-05
k_{15_2}	1.540e-06	k_{32_2}	4.832e-05	k_{48_17}	3.419e-05	d_{36}	3.176e-05
k_{15_4}	0	k_{32_28}	0	k_{48_19}	3.083e-05	d_{37}	4.800e-05

k_{15_13}	0	k_{33_28}	0	k_{49}	8.990e-06	d_{38}	2.874e-05
k_{15_19}	1.000e-06	k_{34_2}	0	k_{49_22}	4.380e-05	d_{39}	5.842e-05
k_{16_2}	0	k_{34_28}	5.052e-05	k_{49_25}	1.000e-05	d_{40}	0
k_{16_11}	0	k34_32	0	k_{49_28}	1.000e-06	d_{41}	0
k_{16_48}	0	k_{35_2}	2.000e-05	k_{50_36}	0	d_{42}	0
k_{17_2}	1.199e-05	k_{35_28}	2.000e-05	a_1	0.002	d_{43}	3.095e-05
k_{18}	4.900e-05	k_{35_30}	4.938e-05	a_2	0.002	d_{44}	2.070e-05
k_{19_2}	1.720e-06	k_{35_32}	1.000e-05	a_3	1.000e-05	d_{45}	1.000e-05
k_{19_6}	2.215e-05	k_{35_36}	0	a_4	2.387e-05	d_{46}	1.000e-05
k_{20_2}	0	k_{35_37}	2.451e-05	a_5	1.000e-05	d_{47}	1.800e-05
k_{20_18}	0	k_{35_39}	0	a_6	2.519e-05	d_{48}	5.000e-05
k_{20_47}	5.000e-05	k_{36_2}	0	d_1	3.790e-05	d_{49}	4.500e-05
k_{20_48}	0	k_{36_28}	7.700e-07	d_2	5.000e-06	d_{50}	0

Note: If the optimal value of the kinetic parameter k_{i_j} is zero, then we will delete the directed edge which indicates biomolecular j regulates biomulecular i in the network. Furthermore, if there is not any edge linking biomocular i, we will delete the node i in the network. At last, if the node i has been deleted in the network, we let the degradation rate d_i is zero in the numerical simulation. Therefore, the optimal value of the kinetic parameter d_i which is zero indicates the node i has been deleted in the network.
