# Supplementary material for: Construction of the influenza A virus infection-induced cell-specific inflammatory regulatory network based on mutual information and optimization
Source: BMC Syst Biol. 2013 Oct 20;7:105. doi: 10.1186/1752-0509-7-105 (PMC4016583; doi:10.1186/1752-0509-7-105)
Supplement: Additional file 5 — This file includes the comparisons between the numerical simulation results and experimental data of proteins in the optimized network. [file 1752-0509-7-105-S5.pdf]

Comparisons between the numerical simulation results and experimental data of proteins in the optimized network (Figure 4 in the main text).

The blue and red lines denote the experiment and similation results, respectively. The stars represent experimental data at each time point. The experimental errors are also plotted as short bars at each time point.

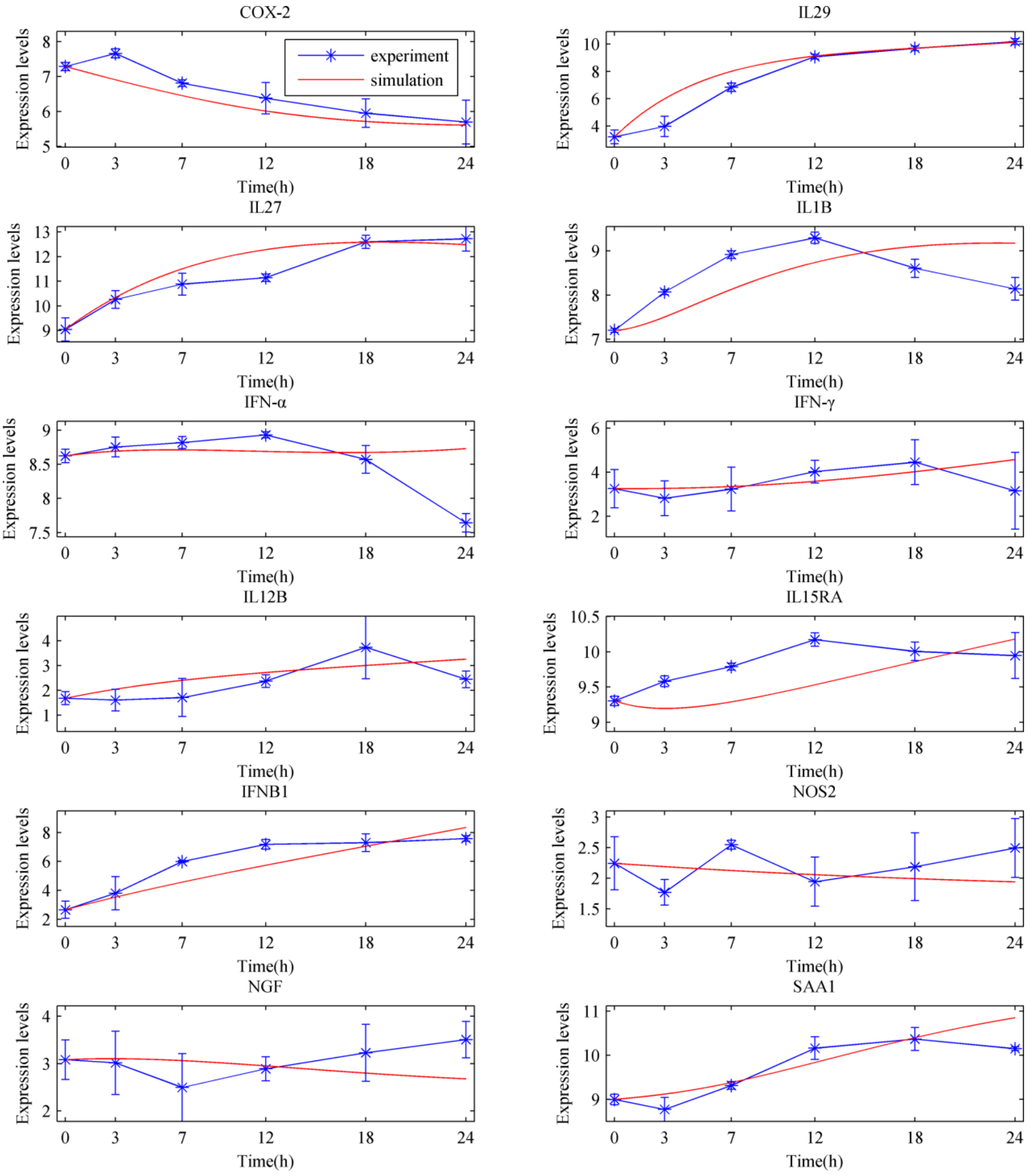

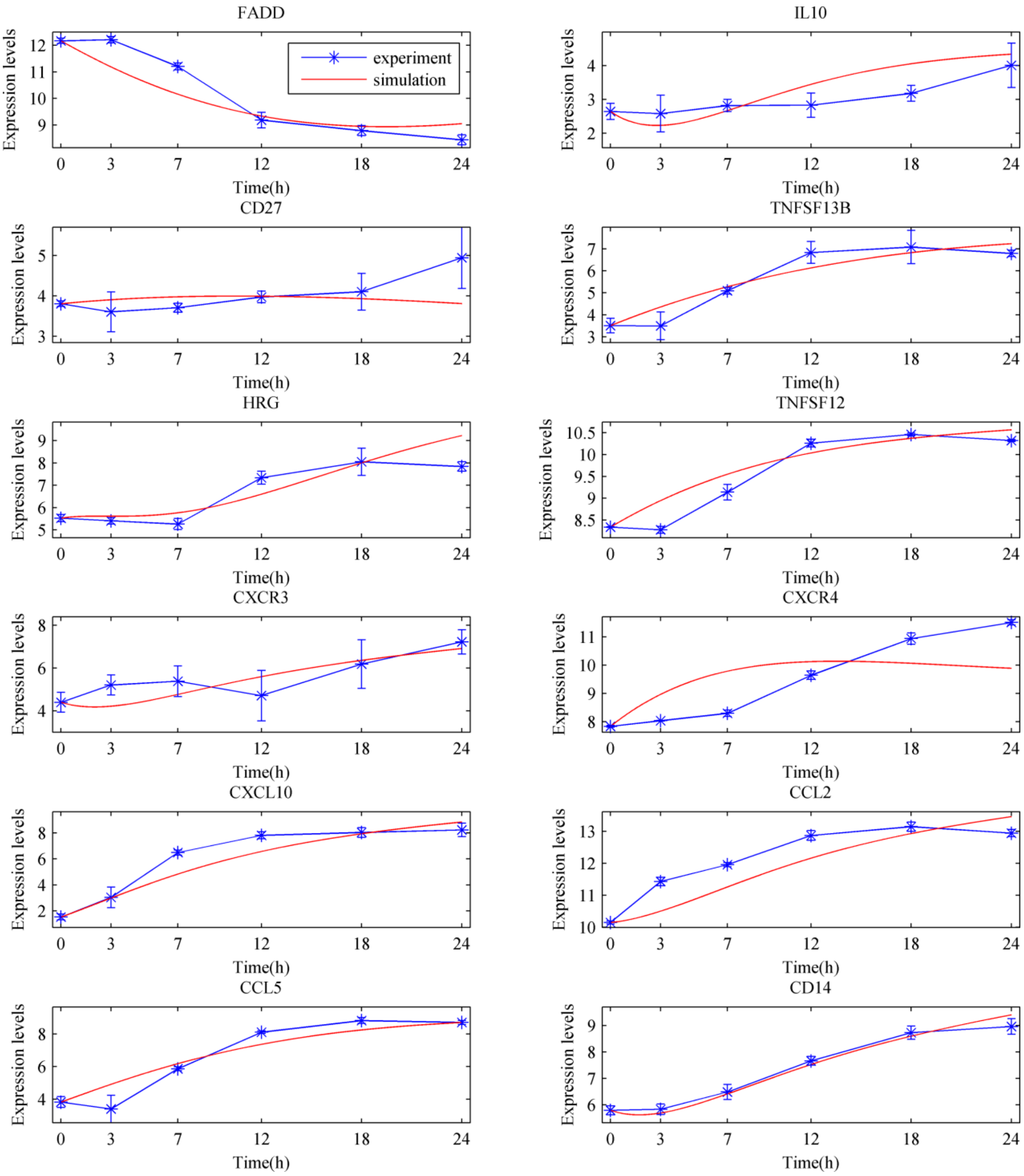
FADD
IL10
CD27
TNFSF13B
HRG
TNFSF12
CXCR3
CXCR4
CXCL10
CCL2
CCL5
CD14
experiment
simulation
Expression levels
Time(h)
0 3 7 12 18 24

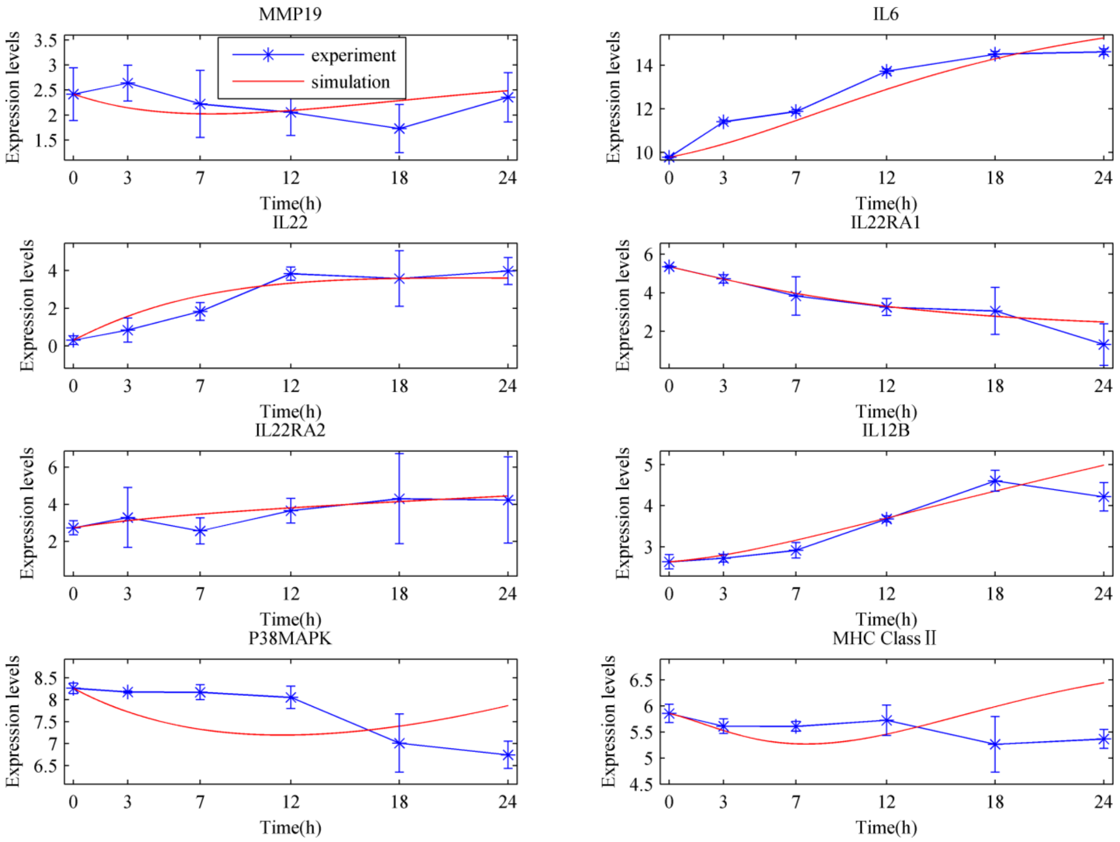

MMP19
experiment
simulation
IL6
IL22
IL22RA1
IL22RA2
IL12B
P38MAPK
MHC Class II
Expression levels
Time(h)
