# Supplementary material for: Construction of the influenza A virus infection-induced cell-specific inflammatory regulatory network based on mutual information and optimization
Source: BMC Syst Biol. 2013 Oct 20;7:105. doi: 10.1186/1752-0509-7-105 (PMC4016583; doi:10.1186/1752-0509-7-105)
Supplement: Additional file 6 — This file includes one additional Table S3 that lists the classification of the interactions in the optimized network. [file 1752-0509-7-105-S6.pdf]

**Table S3:** Classification of the interactions in the optimized network

| Interactions | References |
|---|---|
| IAV→IAV | [1, 2] |
| IAV→COX2 | [3, 4, 5, 6] |
| COX2→NGF | [6, 7] |
| COX2→SAA1 | [6, 7] |
| COX2→TLR3 | [5, 6, 7] |
| COX2→IL32 | [3,6] |
| COX2→IL27 | [3,6] |
| COX2→IL22RA1 | [6, 7] |
| COX2→IL22RA2 | [6, 7] |
| COX2→TNF | [3,4] |
| COX2→CXCR4 | [6, 7] |
| COX2→CCL2 | [6, 7] |
| COX2→TNFSF12 | [6, 7] |
| IL27—\| IAV | [8] |
| NGF→NFκB | [9] |
| TLR3→TLR3 | [10] |
| TLR3→IL29 | [10, 11] |
| TLR3→IFNB1 | [10, 11] |
| TLR3→IL27 | [10, 11, 12] |
| IL32—\| COX-2 | [3] |
| IL32→NOS2 | [13] |
| IL22RA1—IL22 | [14] |
| TNF→CXCL10 | [15, 16] |
| TNF→CCL2 | [17] |
| TNF→TNF | [15, 18] |
| CXCR4→CXCR4 | [19] |
| NOS2—\| IL32 | [13] |
| IL22—IL22RA2 | [14] |
| IL12→IFN-γ | [20] |
| CCL2→IL6 | [21] |
| NFκB→NFκB | [22] |
| NFκB→IFN-β | [22, 23, 24] |
| IL1B→IL1B | [25, 26] |
| IL29→IL29 | [14] |
| IL10→IL10 | [14] |
| MHC Class II→MHC Class II | [27] |
| CD14→IL6 | [28] |
| CD40→CD40 | [29] |
| CD40→IL10 | [29, 30] |
| TNFSF13B→TNFSF13B | [31] |
| IFN-α→IFN-α | [32] |
| IFN-α —\| IAV | [33, 34] |

| | |
|---|---|
| P38MAPK→IFN-α | [35] |
| P38MAPK→IFNB1 | [35] |
| IL12B→IL12B | [36] |
| NGF→IL1B | predicted |
| SAA1→NFκB | predicted |
| SAA1→TLR3 | predicted |
| IL32→IL1B | predicted |
| IL1B→IL32 | predicted |
| TNF→CXCR3 | predicted |
| TNF→MHC Class II | predicted |
| TNF→IL12 | predicted |
| TNF→CD14 | predicted |
| CXCR4→CCL2 | predicted |
| CXCR4→CCL5 | predicted |
| IFN-γ→TNF | predicted |
| NFκB→IL15RA | predicted |
| TNFSF12→CCL2 | predicted |
| TNFSF12→IL6 | predicted |
| IL27→IL1B | predicted |
| CCL2→CCL5 | predicted |
| CCL5→MMP19 | predicted |
| CCL5→TNF | predicted |
| CCL5→IL6 | predicted |
| IL1B→SAA1 | predicted |
| CXCL10→TNF | predicted |
| IL29→IL10 | predicted |
| IL6→TNF | predicted |
| IL10→CCL16 | predicted |
| IL10→HRG | predicted |
| IL10→MHC Class II | predicted |
| IL22→IL10 | predicted |
| IFNB1→P38MAPK | predicted |
| CD14→CCL2 | predicted |
| CD40→MHC Class II | predicted |
| CD40→CD27 | predicted |
| CD40→TNFSF13B | predicted |
| IFN-α→P38MAPK | predicted |
| P38MAPK→IL29 | predicted |
| P38MAPK→FADD | predicted |
| P38MAPK→IL12B | predicted |
| TLR3→IFN-β | false positive interaction |
| TLR3→P38MAPK | false positive interaction |
| IL1B→TLR3 | false positive interaction |
| IL1B→NFkB | false positive interaction |

| | |
|---|---|
| TNF→CCL5 | false positive interaction |
| TNF→IL6 | false positive interaction |
| TNFSF12→TNF | false positive interaction |
| TNFSF12→CCL5 | false positive interaction |

## REFERENCES


[1] König R, Stertz S, Zhou Y, Inoue A, Hoffmann HH, Bhattacharyya S, Alamares JG, Tscherne DM, Ortigoza MB, Liang Y, Gao Q, Andrews SE, Bandyopadhyay S, De Jesus P, Tu BP, Pache L, Shih C, Orth A, Bonamy G, Miraglia L, Ideker T, García-Sastre A, Young JA, Palese P, Shaw ML, Chanda SK: **Human host factors required for influenza virus replication.** Nature 2010, **463:**813-817.

[2] Watanabe T, Watanabe S, Kawaoka Y: **Cellular networks involved in the influenza virus life cycle.** Cell Host Microbe 2010, **7:**427-39.

[3] Li W, Liu Y, Mukhtar MM, Gong R, Pan Y, Rasool ST, chGao Y, Kang L, Hao Q, Peng GQ, Chen YN, Chen X, Wu JG, Zhu Y: **Activation of interleukin-32 pro-inflammatory pathway in response to influenza A virus infection.** PLoS One 2008, **3:**e1985.

[4] Steer SA, Corbett JA: **The role and regulation of COX-2 during viral infection.** Viral Immunol 2003, **16:**447-460.

[5] Chan MC, Cheung CY, Chui WH, Tsao SW, Nicholls JM, Chan YO, Chan WY, Long HT, Poon LL, Guanand Y, Peiris JS: **Proinflammatory cytokine responses induced by influenza A (H5N1) viruses in primary human alveolar and bronchial epithelial cells.** Resp Res 2005, **6:**135.

[6] Lee SM, Cheung CY, Nicholls JM, Hui KP, Leung CY, Uiprasertkul M, Tipoe GL, Lau YL, Poon LL, Ip NY, Guan Y, Peiris JS: **Hyperinduction of cyclooxygenase-2-mediated proinflammatory cascade: a mechanism for the pathogenesis of avian influenza H5N1 infection.** J Infect Dis 2008, **198:**525–535.

[7] Liu L, Li R, Pan Y, Chen J, Li YK, Wu JG, Zhu Y: **High-throughput screen of protein expression levels induced by cyclooxygenase-2 during influenza a virus infection.** Clinica Chimica Acta 2011, **412:**1081-1085.

[8] Liu L, Cao Z, Chen J, Li R, Cao Y, Zhu C, Wu K, Wu J, Liu F, Zhu Y: **Influenza A virus induces interleukin-27 through cyclooxygenase-2 and protein kinase A signaling.** J Biol Chem 2012, **287:**11899-11910.

[9] Wooten MW, Geetha T, Seibenhener ML, Babu JR, Diaz-Meco MT, Moscat J: **The p62 scaffold regulates nerve growth factor-induced NF-kappaB activation by influencing TRAF6 polyubiquitination.** J Biol Chem 2005, **280:**35625-35629.

[10] Arpaia N, Barton GM: **Toll-like receptors: key players in antiviral immunity.** Curr Opin Virol 2011, **1:**447-454.

[11] Abujamra AL, Spanjaard RA, Akinsheye I, Zhao X, Faller DV, Ghosh SK: **Leukemia virus long terminal repeat activates NFkappaB pathway by a TLR3-dependent mechanism.** Virol J 2006, **345:**390-403.

[12] Pirhonen J, Sirén J, Julkunen I, Matikainen S: **IFN-alpha regulates Toll-like receptor-mediated IL-27 gene expression in human macrophages.** J Leukoc Biol 2007, **82:**1185-1192.

[13] Li Q, Yang F, Liu Y, Gong R, Liu L, Feng Y, Hu P, Sun W, Hao Q, Kang L, Wu JG, Zhu Y: **Negative feedback regulation of IL-32 production by iNOS activation in response to dsRNA or influenza virus infection.** Eur J Immunol 2009, **39:** 1019–1024.

[14] Wittea K, Wittea E, Sabata R, Wolk K: **IL-28A, IL-28B, and IL-29: Promising cytokines with type I interferon-like properties.** Cytokine Growth F R 2010, **21:**237-251.

[15] Veckman V, Osterlund P, Fagerlund R, Melén K, Matikainen S, Julkunen I: **TNF-alpha and IFN-alpha enhance influenza-A-virus-induced chemokine gene expression in human A549 lung epithelial cells.** Virol 2006, **345:**96-104.

[16] Hardaker EL, Bacon AM, Carlson K, Roshak AK, Foley JJ, Schmidt DB, Buckley PT, Comegys M, Panettieri RA Jr, Sarau HM, Belmonte KE: **Regulation of TNF-alpha- and IFN-gamma-induced CXCL10 expression: participation of the airway smooth muscle in the pulmonary inflammatory response in chronic obstructive pulmonary disease.** Faseb J 2004, **18:**191-193.

[17] Ho AW, Wong CK, Lam CW: **Tumor necrosis factor-alpha up-regulates the expression of CCL2 and adhesion molecules of human proximal tubular epithelial cells through MAPK signaling pathways.** Immunobiology 2008, **213:**533-44.

[18] Fiedler MA, Wernke-Dollries K, Stark JM: **Inhibition of TNF- α -induced NF-κB Activation and IL-8 Release in A549 Cells with the Proteasome Inhibitor MG-132.** Am J Respir Cell Mol Biol 1998, **19:**259-268.

[19] Puri A, Riley JL, Kim D, Ritchey DW, Hug P, Jernigan K, Rose P, Blumenthal R, Carroll RG: **Influenza virus upregulates CXCR4 expression in CD4+ cells.** Aids Res Hum Retroviruses 2000, **16:**19-25.

[20] Watford WT, Hissong BD, Bream JH, Kanno Y, Muul L, O'Shea JJ: **Signaling by IL-12 and IL-23 and the immunoregulatory roles of STAT4.** Immunol Rev 2004, **202:**139-56.

[21] Liu X, Das AM, Seideman J, Griswold D, Afuh CN, Kobayashi T, Abe S, Fang Q, Hashimoto M, Kim H, Wang X, Shen L, Kawasaki S, Rennard SI: **The CC chemokine ligand 2 (CCL2) mediates fibroblast survival through IL-6.** Am J Respir Cell Mol Biol 2007, **37:**121-8.

[22] Ludwig S, Planz O: **Influenza viruses and the NF-kappaB signaling pathway - towards a novel concept of antiviral therapy.** Biol Chem 2008, **389:**1307-1312.

[23] Schmolke M, Viemann D, Roth J, Ludwig S: **Essential impact of NF-kappaB signaling on the H5N1 influenza A virus-induced transcriptome.** J Immunol 2009, **183:**5180-9.

[24] Pauli EK, Schmolke M, Wolff T, Viemann D, Roth J, Bode JG, Ludwig S: **Influenza A virus inhibits type I IFN signaling via NF-kappaB-dependent induction of SOCS-3 expression.** PLoS Pathog 2008, **4:**e1000196.

[25] Hiscott J, Kwon H, Génin P: **Hostile takeovers: viral appropriation of the NF-kB pathway.** J Clin Invest 2001, **107:**143–151.

[26] Elinav E, Strowig T, Henao-Mejia J, Flavell RA: **Regulation of the antimicrobial response by NLR proteins.** Immunity 2011, **34:**665-79.

[27] Sinnathamby G, Eisenlohr LC: **Presentation by recycling MHC class II molecules of an influenza hemagglutinin-derived epitope that is revealed in the early endosome by acidification.** J Immunol 2003, **170:**3504-3513.

[28] Baumann CL, Aspalter IM, Sharif O, Pichlmair A, Blüml S, Grebien F, Bruckner M, Pasierbek P, Aumayr K, Planyavsky M, Bennett KL, Colinge J, Knapp S, Superti-Furga G: **CD14 is a coreceptor of Toll-like receptors 7 and 9.** J Exp Med 2010, **207:**2689-2701.

[29] Foey AD, Feldmann M, Brennan FM: **CD40 ligation induces macrophage IL-10 and TNF-alpha production: differential use of the PI3K and p42/44 MAPK-pathways.** Cytokine 2001, **16:**131-142.

[30] D'Amico G, Vulcano M, Bugarin C, Bianchi G, Pirovano G, Bonamino M, Marin V, Allavena P, Biagi E, Biondi A: **CD40 activation of BCP-ALL cells generates IL-10-producing, IL-12-defective APCs that induce allogeneic T-cell anergy.** Blood 2004, **104:**744-51.

[31] Mackay F, Browning JL: **BAFF: A fundamental survival factor for B cells.** Nat Rev Immunol 2002, **2:**465-475.

[32] Pauli EK, Schmolke M, Wolff T, Viemann D, Roth J, Bode JG, Ludwig S: **Influenza A virus inhibits type I IFN signaling via NF-kappaB-dependent induction of SOCS-3 expression.** PLoS Pathog 2008, **4:**e1000196.

[33] Cheney KM, McKnight A: **Interferon-alpha mediates restriction of human immunodeficiency virus Type-1 replication in primary human macrophages at an early stage of replication.** PLoS One 2010, **5:**e13521.

[34] Goodbourn S, Didcock L, Randall RE: **Interferon: cell signalling, immune modulation, antiviral response and virus countermeasures.** J Gen Virol 2000, **81:**2341-2364.

[35] Hui KP, Lee SM, Cheung CY, Ng IH, Poon LL, Guan Y, Ip NY, Lau AS, Peiris JS: **Induction of proinflammatory cytokines in primary human macrophages by influenza A virus (H5N1) is selectively regulated by IFN regulatory factor 3 and p38 MAPK.** J Immunol 2009, **182:**1088-1098.

[36] Monteiro JM, Harvey C, Trinchieri G: **Role of interleukin-12 in primary influenza virus infection.** J Virol 1998, **72:**4825-4831.
