# Supplementary material for: Construction of the influenza A virus infection-induced cell-specific inflammatory regulatory network based on mutual information and optimization
Source: BMC Syst Biol. 2013 Oct 20;7:105. doi: 10.1186/1752-0509-7-105 (PMC4016583; doi:10.1186/1752-0509-7-105)
Supplement: Additional file 8 — This file includes the comparison study for the advantage of involving non-linear items in the model. [file 1752-0509-7-105-S8.doc]

This file includes the comparison study for the advantage of involving non-linear items in the model.

The selection of a suitable model for network description is a non-trivial component of network inference. Most of model-based studies for inferring networks are based on linear ODEs models. However, complex dynamic behaviors such as the emergence of multiple steady states (e.g. healthy or disease state), cannot explained by simple linear systems. Instead, the regulatory interactions in real biological networks always are non-linear.

To show the advantage of the non-linear ODEs model in network inference, in this study, we also built up a linear ODEs model of the network. Specifically, according to the non-linear ODEs model in the Additional file 3, we directly transformed the non-linear terms of the model into linear terms. For example, the linear forms of Eqs. S1 and S2 are as follows:

$$\begin{matrix} \frac{dx_{1}}{dt}=k_{1}x_{1}-a_{1}x_{4}-a_{2}x_{7}-d_{1}x_{1}\text{ (S1')} \\ \frac{dx_{2}}{dt}=k_{2\_1}x_{1}-a_{3}x_{4}-a_{4}x_{5}-d_{2}x_{2}\text{ (S2')} \end{matrix}$$

Similarly, other non-linear terms of the model can be transformed into the linear terms and then a linear ODEs model of the network was constructed. Similar to the methods in the main text, we estimated all parameters in the linear ODEs by the DE algorithm. The DE algorithm was carried out ten times, and the best parameter set was obtained. Based on the optimal parameters, we computed the average relative errors (AREs）in the linear ODEs model.

Figure S1 gives the comparsion of the distributions of AREs between linear and non-linear models, which shows that the AREs in the linear model exhibited significantly higher values than those in the non-linear model (P-value<0.001, a paired Wilcoxon rank sum test). Combining Figure S1 and Figure 6 of main text, we can find that the non-linear ODEs model performs better than the linear ODEs model in network inference.


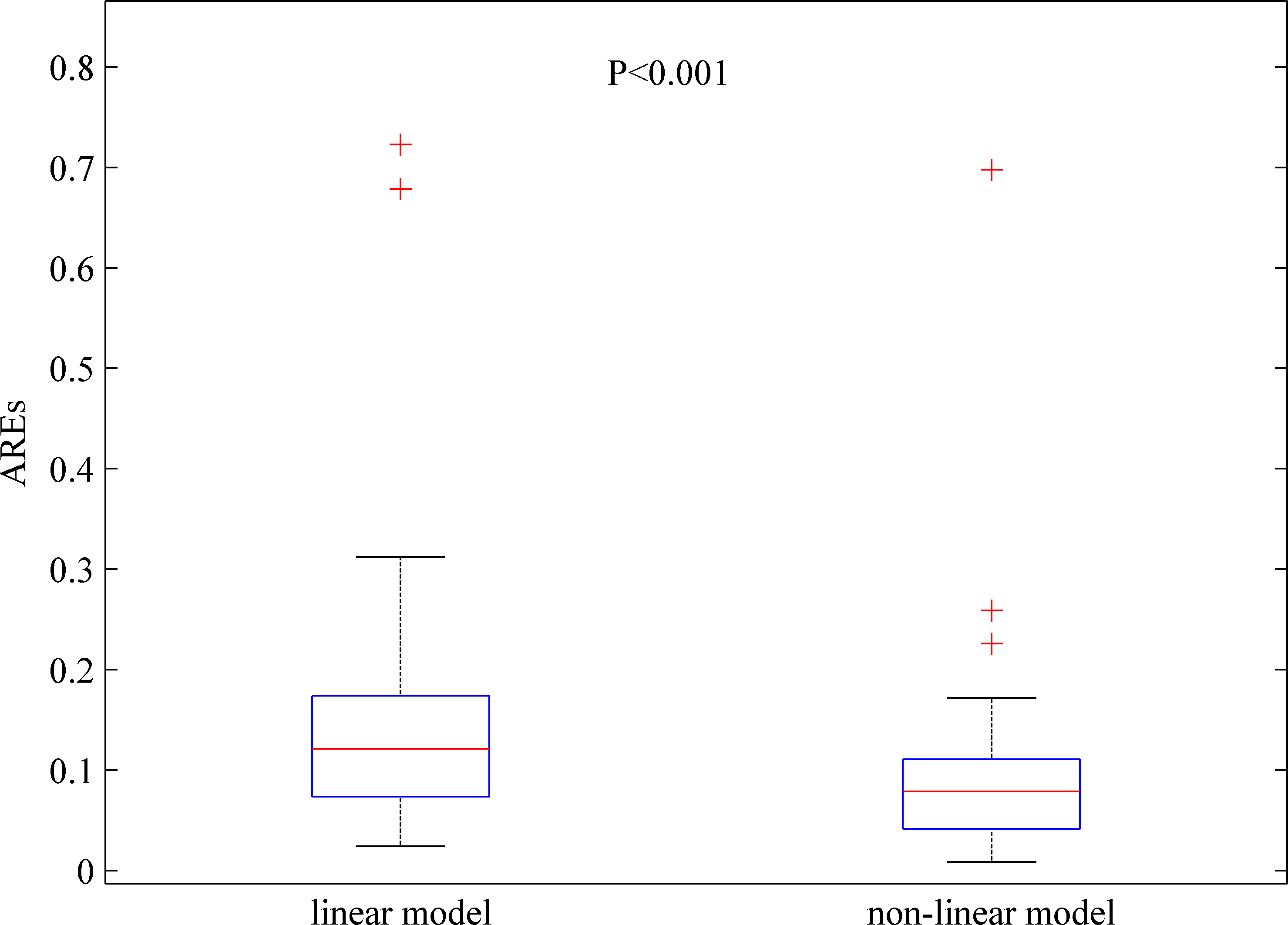


**Figure S1.** Comparison of the average relative errors (AREs) between linear and non-linear models. P-value is from a paired Wilcoxon rank sum test. The AREs in the linear model exhibited significantly higher values than those in the non-linear model (P-value<0.001).
