# Supplementary material for: Construction of the influenza A virus infection-induced cell-specific inflammatory regulatory network based on mutual information and optimization
Source: BMC Syst Biol. 2013 Oct 20;7:105. doi: 10.1186/1752-0509-7-105 (PMC4016583; doi:10.1186/1752-0509-7-105)

This file includes the initial inflammatory rerulatory network by using Ingenuity Pathway Analysis (IPA) software based on the data from Gene Expression Omnibus (GEO) database under the GEO accession number GSE28166 and the website for all uploaded source codes and data (http://maths.whu.edu.cn/teach/lj/?title=%E9%82%B9%E7%A7%80%E8%8A%AC).

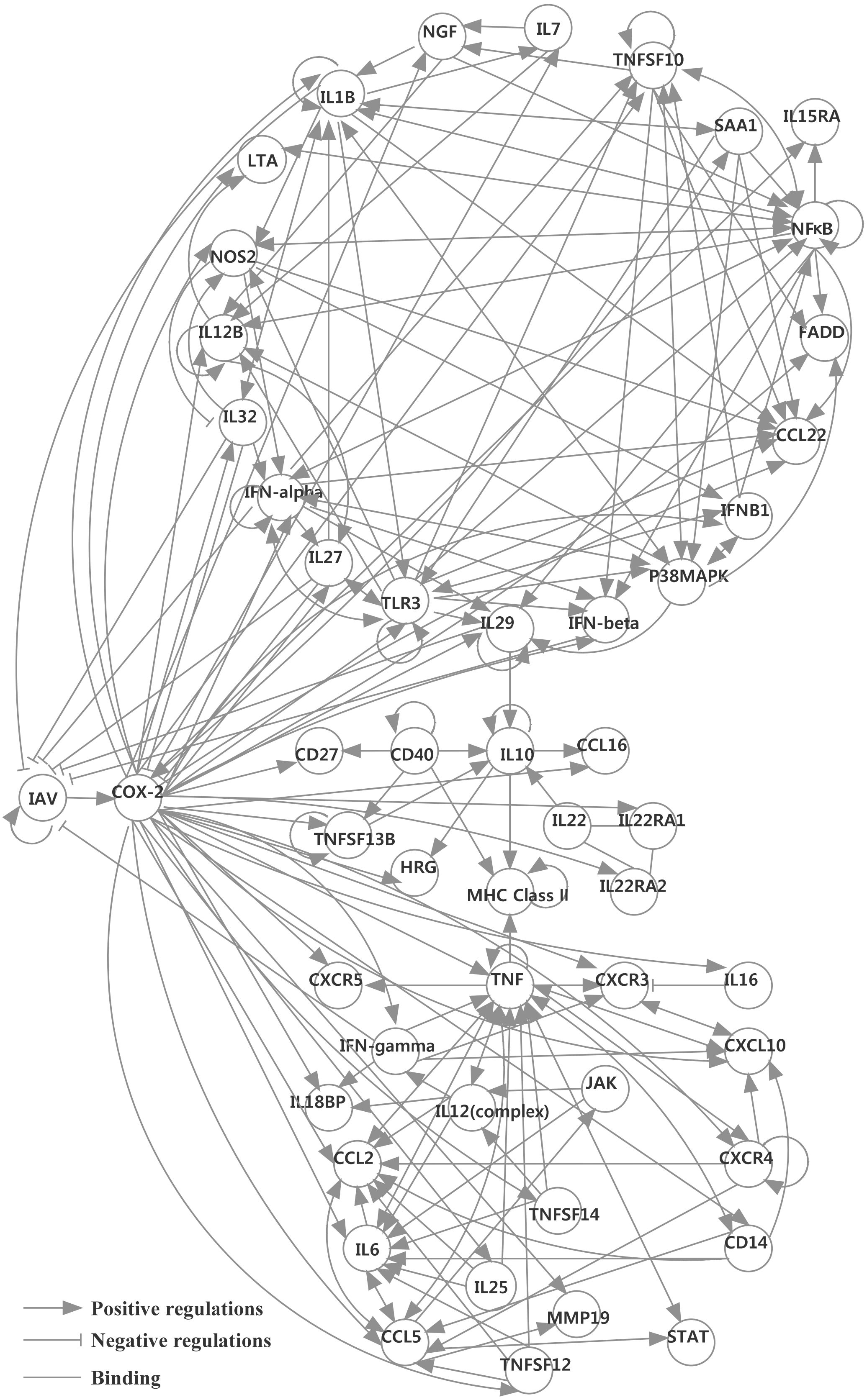

Supplement: Additional file 1 — This file includes the initial inflammatory regulatory network by using Ingenuity Pathway Analysis (IPA) software based on the data from Gene Expression Omnibus (GEO) database under the GEO accession number GSE28166 and the website for all uploaded source codes and data. [file 1752-0509-7-105-S1.pdf]
